# New insights into the N-glycomes of *Dictyostelium* species

**DOI:** 10.1016/j.bbadva.2025.100142

**Published:** 2025-01-16

**Authors:** Alba Hykollari, Daniel Malzl, Chunsheng Jin, Carina Eschenbach, Kristína Kianičková, Iain B.H. Wilson, Katharina Paschinger

**Affiliations:** a Department für Chemie, Universität für Bodenkultur, 1190 Wien, Austria; b Department für Interdisziplinäre Lebenswissenschaften, Veterinärmedizinische Universität, 1160 Wien, Austria; c Institutionen för biomedicin, Göteborgs universitet, 405 30 Göteborg, Sweden

**Keywords:** Dictyostelia, N-glycan, Fucose, Galactose, Phosphate, Sulphate

## Abstract

Dictyostelia are cellular slime molds, a group of Amoebozoa, that form multicellular fruiting bodies out of aggregating cells able of differentiating into resistant spore forms. In previous studies on *Dictyostelium discoideum*, it was demonstrated that their N-glycans, as in most eukaryotes, derive from the Glc_3_Man_9_GlcNAc_2_-PP-Dol precursor; however, unique glyco-epitopes, including intersecting GlcNAc, core α1,3-fucosylation, sulphation and methylphosphorylation, were detected. In the present study, we have examined the N-glycans of two other *Dictyostelium* species, *D. purpureum,* whose genome is also sequenced, and *D. giganteum*. The detailed glycomic analysis of their fruiting bodies was based on isomeric separation of the glycan structures by HPLC, followed by mass spectrometry in combination with enzymatic digests and chemical treatments. Two features absent from the 'model' dictyostelid *D. discoideum* were found: especially in *D. purpureum,* a long linear galactose arm β1,4-linked to the β1,4-*N*-acetylglucosamine on the 'lower' A-branch of its oligo-mannosylated structures could be identified. In contrast, neutral N-glycans with multiple fucose residues attached to terminal mannoses were found in *D. giganteum*. All three species have common modifications on their anionic N-glycans: while (methyl)phosphorylated residues are always associated with terminal mannose residues, the sulphation position differs. While *D. discoideum* has 6-sulphation of subterminal mannose residues, *D. giganteum* and *D. purpureum* may rather have 2-sulphation of core α1,6-mannose. Overall, we have discovered species-specific glycan variations and our data will contribute to future comparative and functional studies on these three species within the same genus.

## Highlights

• Dictyostelium giganteum and D. purpureum express species-specific N-glycans.
• Novel galactosylated and fucosylated glycan motifs were found.
• 20 % of the their glycans carry methylphosphate and/or sulphate modifications.

## 1 Introduction

The Amoebozoa are simple life forms, closely related to the opisthokonts including opportunistic unicellular and obligate parasitic species, the true slime molds (*Physarium*) and the so-called social amoeba. Advanced genomic species analyses could confirm their monophyly and, based on phylogenetic studies, four major groups or families could be proposed [1,2]. The model organism *Dictyostelium discoideum* is the best-known species within the 4th group, i.e., the Dictyostelids. As its lifecycle involves both unicellular and aggregated states, it shows characteristics of both protists and metazoa. The social amoeba *D. discoideum* has been used as a model system for over 70 years, providing information on basic cell and developmental biology with similarities between the cell motility and signalling systems of protists and metazoa as well as the facultative multicellular life-style of the social amoeba [3]. Previous genomic comparisons between Dictyostelid species suggests that, although their genomes are similar in size and content, some gene families have markedly diverged [4]. Amongst these related species, *D. purpureum* produces large fruiting bodies with single sori containing spores, held by a single cellular stalk [5,6], while *D. giganteum* is studied primarily for its alternative sexual life cycle and mating type formation [7,8]. Due to their morphologically different stalks and spore coats, both these species are attractive for stage-specific comparisons.

Beside comparative genomics, studies of proteins and their modifications within Dictyostelid species are rare as the focus has been on *D. discoideum* [[9], [10], [11], [12], [13], [14]]. In terms of glycomics, the initial steps of N-glycan biosynthesis in this species have been shown to be conserved in comparison to yeast, plants and animals. In the one cross-species profiling study to date, the N-glycan compositions were found to vary between species and between developmental stages, whereby differences in glyco-epitopes are well documented in *D. discoideum* [11,15,16]. Thereby, after the initial transfer of Glc_3_Man_9_GlcNAc_2_ to proteins, subsequent processing in the Golgi yields a diverse spectrum of N-glycan structures that play roles in the function of the modified proteins outside Golgi cisternae; for instance, the developmental function of the TgrB1- and TgrC1 adhesion complex is modulated by altered N-glycosylation [17,18]. On the other hand, other than their detection [[19], [20], [21]], relatively little is known about O-glycosylation and phosphoglycosylation events in the secretory pathway in Dictyostelids, but a cytosolic O-glycan has an important role in oxygen sensing [22]; furthermore, glycosylphosphatidylinositol membrane anchored proteins have been described [23]. Dictyostelids also produce polysaccharides, including a galactose-rich heteropolysaccharide and cellulose as components of the spore coat [14,20].

Over the years, a number of different approaches have been used to analyse the N-glycans of *D. discoideum*, including radiolabelling, NMR, liquid chromatography and mass spectrometry [[24], [25], [26], [27]]. Particularly, the mix of HPLC and MALDI-TOF MS has revealed the exact structures of N-glycans from *D. discoideum* wild-type and mutant strains and some 100 or more 'glyco-variants' have been established [28,29]. While the neutral N-glycome of *D. discoideum* displays core α1,3-fucosylation as well as bisecting and intersecting *N*-acetylglucosamine residues [15,30], the anionic structures have been especially challenging to analyse. Phosphorylated epitopes for example, were primarily identified in *D. discoideum* as methylphosphate, decorating the terminal mannose residues of N-glycan branches [28,[31], [32], [33]]. At the cellular stage, up to three methylphosphate residues were identified, whereas the un-methylated phosphate form is very rare and *N*-acetylglucosamine-1-phosphate (i.e., a phosphodiester), an intermediate product of the *gpt1* enzyme, is obvious rather on N-glycans of a glucosidase-deficient strain [29,34]. Sulphation, probably 6-linked, predominantly occurs on subterminal mannose residues and some glycans in *D. discoideum* contain both methylphosphate and sulphate which means they are rather resistant to glycosidase digestions [28,35].

In this study we present the N-glycomes of the developed fruiting bodies of two Dictyostelid species other than *D. discoideum*, specifically *D. giganteum* and *D. purpureum*. The modifications found include not only methylphosphate and sulphate, but also some unexpected modifications, i.e., multiple terminal galactose and fucose residues. Overall, in this comparative study of two non-axenic wild-type species, we define that there are species-specific as well as shared glyco-epitopes indicative of differences in the occurrence and expression of glycosyltransferase genes.

## 2 Experimental procedures

The *Dictyostelium purpureum* (DBS0266810, B12A) and *Dictyostelium giganteum* (DBS0302499, IAS) strains were obtained from the Dictyostelium Stock Centre, and grown on SM5 agar plates on *E. coli* strain OP50 according to standard techniques. Fruiting bodies (1–2 g wet weight), were collected directly from the plates, washed free of bacteria and spores were filtered with a 70 µm cell strainer prior to heat treatment in water for 10 min. After cooling, the material was homogenised by sonication and proteolysed with pepsin in 5 % formic acid (v/v) over night at 37 °C, prior to purification of the (glyco)peptides and release of N-glycans with PNGase A (NEB) and F (Roche) as previously published [36]. After release, the flow-through from a second Dowex AG50 Wx8 chromatography step (***Supplementary Figure 1A***) was subject to solid phase extraction using non-porous graphitised carbon (300 µl ENVI™ Carb, Supelco, Sigma-Aldrich). Elution with 40 % acetonitrile and then 40 % acetonitrile containing 0.1 % trifluoroacetic acid was performed to respectively enrich the neutral and anionic N-glycans. The enriched pools were then separately labelled with 2-aminopyridine at the reducing terminus prior to subsequent RP-HPLC separation and MALDI TOF MS/MS analysis [37]. MALDI-TOF MS of the N-glycome pools was used for quality control before and after labelling (***Supplementary Figure 1B***).

### 2.2 Reversed phase chromatography of N-glycans

RP-HPLC (Shimadzu Nexera UPLC) separation using an Ascentis Express RP-amide column (150 × 4.6 mm, 2.7 µm; Sigma Aldrich, calibrated in terms of glucose units with a pyridylaminated oligoglucose standard) was employed with a gradient of 30 % (v/v) methanol (buffer B) in 100 mM ammonium acetate, pH 4 (buffer A) being applied at a flow rate of 0.8 ml/min as follows: 0–4 min, 0 % B; 4–14 min, 0–5 % B; 14–24 min, 5–15 % B; 24–34 min, 15–35 % B; 34–35 min, return to starting conditions.

### 2.3 MALDI-TOF MS/MS of N-glycans

HPLC fractions were dried and reconstituted in deionized water and analysed by MALDI-TOF MS using 3 mg/ml 6-aza-2-thiothymine as matrix, whereby the samples were dried under vacuum on a steel target prior to application of the matrix (dissolved in 50 % ethanol) and drying once again. MS and MS/MS (laser-induced dissociation) spectra were generated using either an Autoflex or a RapifleX Speed MALDI TOF/TOF in positive and negative reflectron ion modes. To distinguish multiply-sulphated glycans, 20 mM sodium acetate was added to the matrix. Spectra were processed with the manufacturer's software (Bruker Flexanalysis 3.3.80) using the SNAP algorithm with a signal/noise threshold of 6 for MS (unsmoothed) and 3 for MS/MS (smoothed four times) and thereafter analysed manually. Typically 4000 shots were collected for MS and 10,000 for MS/MS modes (see supplementary information). A selected pyridylaminated N-glycan was analyzed by online LC-MS/MS using a 10 cm x 150 µm I.D. column (5 µm porous graphitized carbon) coupled to an LTQ ion trap mass spectrometer (Thermo Scientific). Glycans were eluted using a linear gradient from 0 to 40 % acetonitrile in 10 mM ammonium bicarbonate over 40 min at a flow rate of 10 µl/min and detected in negative ion mode with an electrospray voltage of 3.5 kV, capillary voltage of ∼33.0 V and temperature of 300 °C. MS^n^ fragmentation was performed by collision induced dissociation (CID), energy set at 30 % and air as sheath gas. The data was processed with the Xcalibur software (Version 2.0.7, Thermo Scientific). The N-glycans were manually annotated using the nomenclature of Domon and Costello [38].

### 2.4 Chemical and glycosidase treatments

Phosphomono- and di-ester bonds as well as fucose on pyridylaminated N-glycans were cleaved with hydrofluoric acid (48 % HF) on ice at 4 °C for 36 h prior to evaporation under vacuum. For removal of phosphomonoesters, we also used shrimp alkaline phosphatase (0.3 U, Fermentas) in 20 mM ammonium bicarbonate/carbonate buffer, pH 8, overnight at 37 °C. Mannosidase and galactosidase digestions of pyridylaminated N-glycans were performed at 37 °C in a final concentration of 50 mM ammonium acetate buffer (pH 5.0) using either *Canavalia ensiformis* α-mannosidase (jack bean; 0.02 U, Sigma-Aldrich) [39], *Aspergillus saitoi* α1,2-specific mannosidase (Prozyme) [40], *Xanthomonas* mannosidase II and III (respectively α1,6 and α1,2/3-specific; New England Biolabs) [41] and *Aspergillus oryzae* β1,4-specific galactosidase (Sigma) [42]. Hexosaminidase or fucosidase digestions of N-glycans were performed with jack bean hexosaminidase (Sigma-Aldrich) [43], *Streptococcus plicatus* β1,3/4-specific hexosaminidase/chitinase (New England Biolabs) [44], *Xanthomonas* β1,2-specific N-acetylglucosaminidase (New England Biolabs) [41] or bovine kidney α-fucosidase (Sigma) in 50 mM ammonium acetate pH 5.5 overnight at 37 °C. Endomannosidase digestions were performed using purified recombinant *Bacteroides xylanisolvens* BxGH99 in 50 mM ammonium acetate (pH 6.5) buffer overnight at 37 °C [34,45]. In the case of sequential enzymatic treatments, the first enzyme was heat inactivated (5 min, 95 °C) prior to addition of the second enzyme. Typically, 1 µl of any HPLC fraction (equivalent to 2–10 mV fluorescence; i.e., in the range 2–10 pmol) was used for these digests performed in PCR tubes, an aliquot of which (0.5 µl out of the total volume of 3 µl) was afterwards measured directly with MALDI-TOF MS without any further purification (except when RP-HPLC was used to isomerically discriminate the digested products).

## 3 Results

### 3.1 Analytical approach for comparative N-glycome analysis of D. purpureum and D. giganteum

The detailed N-glycome analysis of both species was enabled, on the one hand, by the pre-fractionation on NPGC to enrich neutral and anionic N-glycans. On the other hand, labelling of the enzymatically released glycans with 2-aminopyridine enables fluorescence detection during separation of isomeric glycans using a fused core RP-amide HPLC column and improves MALDI TOF MS sensitivity, while increasing the intensity of Y-fragment ions including the core chitobiose. As we have published for *Dictyostelium discoideum* and other invertebrate species [36,46,47], N-glycans were analysed in positive and negative ion modes.

**Fig. 1:**
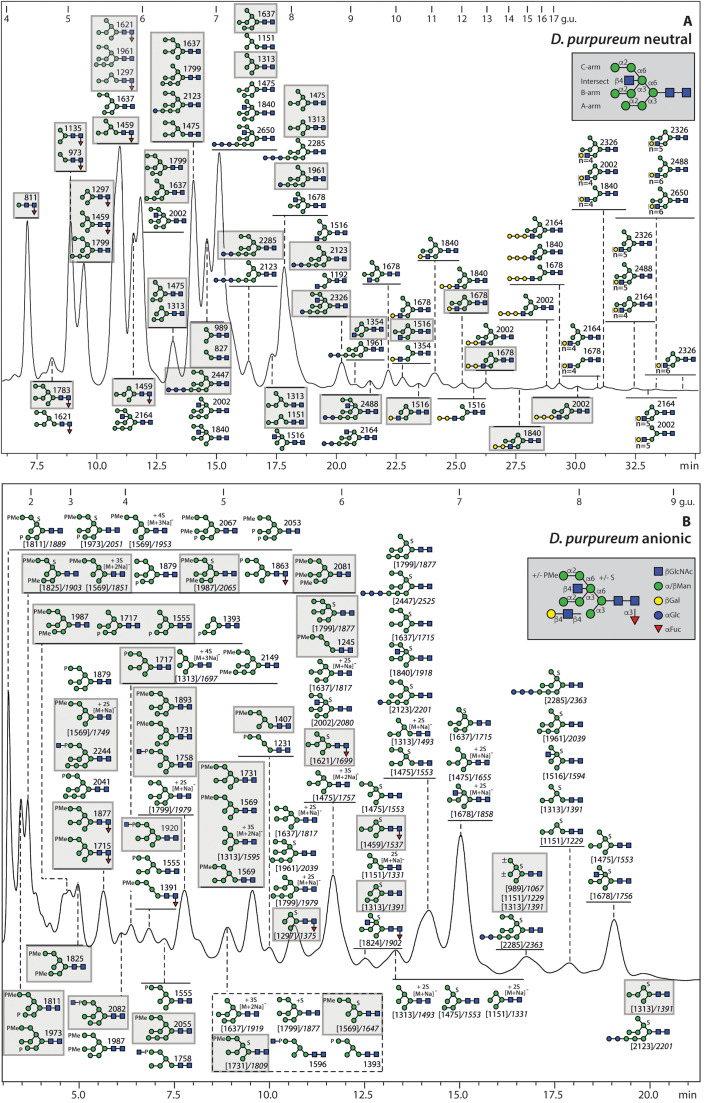
**RP-HPLC analysis of pyridylaminated N-glycans from *D. purpureum*. (A and B)** RP-amide HPLC chromatograms of pyridylaminated neutral and anionic PNGase F and A-released N-glycans from the fruiting bodies of *D. purpureum*. Enrichment by NPGC-SPE resulted in neutral and anionic pools prior to pyridylamination (see Supplementary Figure 1 for the overall MALDI-TOF MS) and RP-HPLC. The reverse phase column was calibrated in terms of glucose units (g.u.). Structures are annotated according to the nomenclature of the Symbolic Nomenclature for Glycomics (circles: mannose in green and galactose in yellow; squares: N-acetylglucosamine in blue; triangle: fucose in red; S suphate; P phosphate; Me methyl groups; see inset) on the basis of retention time and MS/MS with or without chemical/enzymatic treatments; *m/z* values are shown as [*M* + *H*]^+^ or in italics, for sulphated glycans, [M-H]^-^ or [M-nH+(n-1)Na]^-^. Sulphated structures are detected only in negative mode, whereby monosulphated glycans were detected as [M-H]^-^ and di-, tri- and tetrasulphated ones in sodiated form; in positive mode, the sulphate is lost in source (*m/z* values in square brackets). Structures common to both *D. purpureum* and *D. giganteum* are highlighted with grey squares; structures are shown in accordance with the observed abundance in the individual fractions (most abundant uppermost). Oligomannosidic glycans dominate the neutral pool; in addition, there are early-eluting core α1,3-fucosylated Hex_1–7_HexNAc_2_Fuc_1_ and late eluting Hex_3–12_HexNAc_3_ glycans. The anionic pool contains many of the same oligomannosidic-type glycans modified mainly with methylphosphate or sulphate, but lacks galactosylated forms. The terms A-arm, B-arm, C-arm and intersect are defined for an example Hex_8_HexNAc_3_ glycan.

**Fig. 2:**
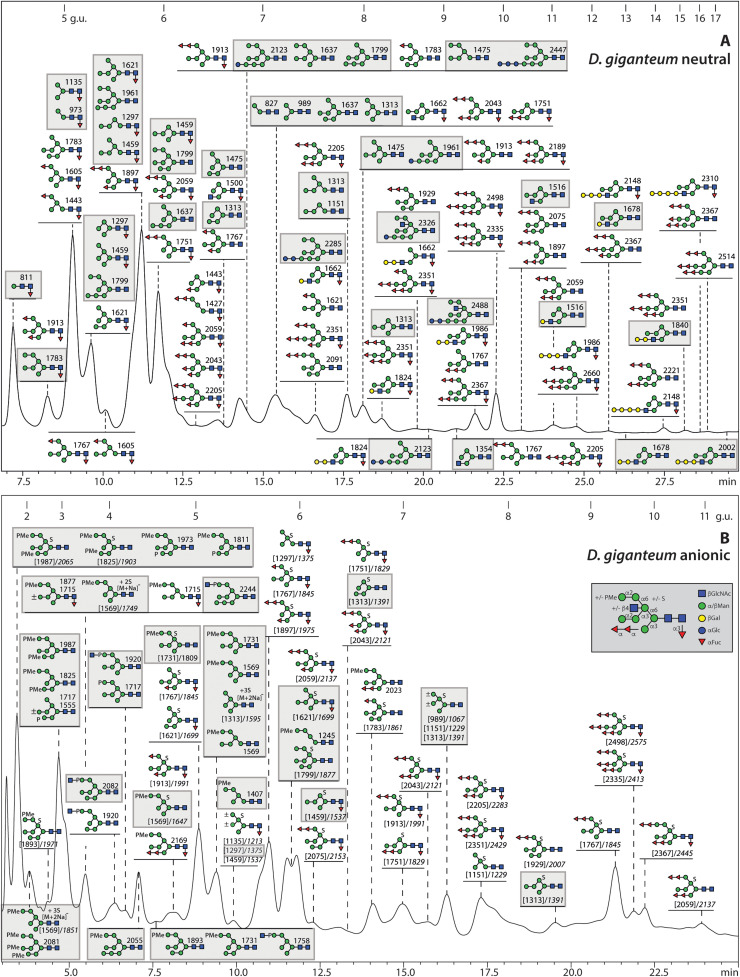
**RP-HPLC fractionation of neutral and anionic pyridylaminated N-glycans from***D. giganteum***. (A and B)** RP-amide HPLC chromatograms of pyridylaminated neutral and anionic PNGase F and A-released N-glycans from the fruiting bodies of *D. giganteum* (see Supplementary Figure 1 for the overall MALDI-TOF MS). As in Fig. 1, the chromatogram is annotated with glucose units and each glycan is shown as a symbolic structure (see also inset) together with the relevant *m/z* value. Structures common to both *D. purpureum* and *D. giganteum* are highlighted with grey squares, especially including oligomannosidic and monofucosylated glycans. In contrast to *D. discoideum* and *D. purpureum,* more glycans are core fucosylated with up to six further fucose residues substituting terminal mannoses. The anionic pool contains many of these fucosylated glycans modified with methylphosphate or sulphate, but the presence of antennal fucose correlates with a lower degree of sulphation, i.e., only three multisulphated structures as compared to seventeen in *D. purpureum*. The oligomannosidic methylphosphorylated structures are shared in both species, but multifucosylated monosulphated ones occur only in *D. giganteum*.

The initial N-glycome spectra for *D. purpureum* and *D. giganteum* indicated a range of mannosidic Hex_2–12_HexNAc_2_ structures. In *D. purpureum* monofucosylated masses were detected, but a higher degree of fucosylation (Hex_6–7_HexNAc_2_Fuc_2–7_) was apparent in the neutral pool of *D. giganteum* glycans; in the anionic pools, the dominant masses in *D. giganteum* corresponded to fucosylated forms of Hex_5–6_HexNAc_2_S, while Hex_4–12_HexNAc_2_S and traces of methylphosphorylated glycans were observed in *D. purpureum* (***Supplementary Figure 1B***). Subsequently, the fractionation of the glycans on the RP-Amide column was followed by MALDI TOF MS/MS and selected chemical or enzymatic treatments to assign the structures (Figs. 1 *and* 2). The more detailed off-line LC-MS approach confirmed the high degree of fucosylation of the N-glycome of fruiting bodies from *D. giganteum* (Fig. 2 and ***Supplementary Figure 1C***), as compared to *D. purpureum.* Indeed, the latter species, akin to *D. discoideum*, had maximally one fucose on glycans with compositions of Hex_2–7_GlcNAc_2_Fuc_1_ (Fig. 1 and ***Supplementary Figure 1C***). The fucose was α1,3-linked to the core GlcNAc as confirmed by HF sensitivity (see below).

In both species, some simple mannosidic glycans (Hex_4–6_HexNAc_2_) were detected in various isoforms. Comparisons can be partly made to the earlier studies on 'classical' C18 columns as compared to the RP-amide column used here [48,49], whereby the B-arm is associated with longer retention times, e.g., the Man_6_GlcNAc_2_ structure eluting at 8 g.u.. Together with relative intensities of the Y fragment ions for the fractionated oligomannosidic glycans reflecting their A arms, the effect of α1,2-mannosidase treatment often indicated a long B-arm (for a definition, see inset in Fig. 1***A***) and occasionally a lack of the lower α1,3-mannose. Indeed, there are multiple isomers of these structures, e.g., four isomers each of Man_5–6_GlcNAc_2_ in *D. purpureum*, distinguishable by retention time and MS/MS fragmentation (***Supplementary Figure 2A-J***). In addition, glucosylated and intersected glycans were rather pronounced in *D. purpureum* (***Supplementary Figure 2K-T***). The approximately 140 masses representing 260 different glycans are listed in ***Supplementary*** ***Table 1****.*

### 3.2 (Methyl)Phosphorylation of mannose is a major anionic modification in Dictyostelium species

Previous studies on the anionic N-glycans from the cellular *D. discoideum* strain Ax3 indicated that these were often modified with methylphosphate linked to terminal α1,2-mannose residues, whereas non-methylated phosphate and GlcNAc-1-phosphate were rare [29,50]. As in those analyses, we employed sensitivity/resistance to the broadly-specific jack bean α-mannosidase, hydrofluoric acid and phosphatase treatments in combination with MALDI TOF MS/MS fragments to define the position of these phospho-epitopes. In the current study, all three phosphate modifications were found, primarily on Hex_5–9_HexNAc_2_ structures.

**Fig. 3:**
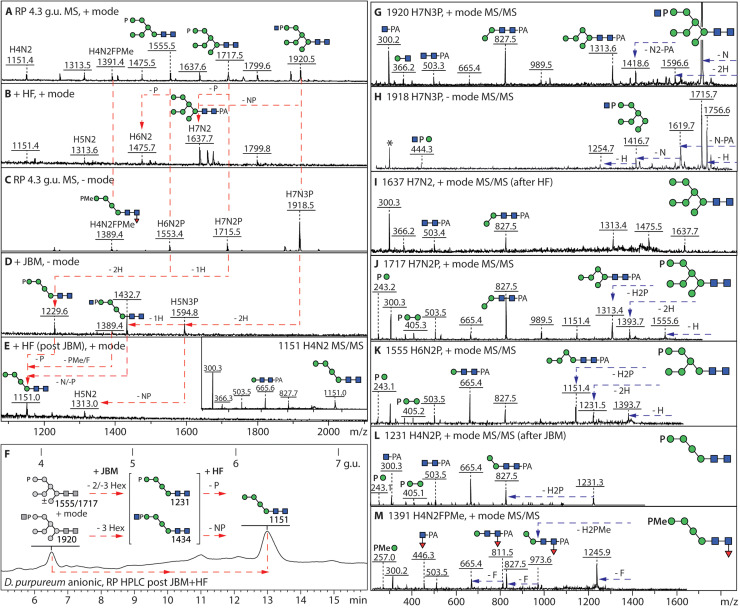
**Phospho-mono- and diesters as modifications of mannose residues of *D. purpureum* anionic N-glycans*.* (A, C)** The 4.3 g.u. fraction of the *D. purpureum* anionic pool was subject to positive and negative mode MALDI-TOF MS showing various phosphorylated, methylphosphorylated and phosphodiester-modified structures. **(B)** Hydrofluoric acid treatment resulted in loss of the phosphoester modifications. **(D-F)** Jack bean α-mannosidase digestion resulted in products of Man_4–5_GlcNAc_2–3_P, converted subsequently to Man_4–5_GlcNAc_2_ upon HF treatment and the combined treatment caused a change in RP-amide elution time correlating with the annotated isomer; alterations are shown by red arrows. **(G-I)** Positive and negative mode MS/MS of the phosphodiester modified Man_7_GlcNAc_3_P structure shows the GlcNAc-1-P of the “upper” C arm, whereby the negative ion mode *m/z* 444 and 1416 B1 and B4 fragments (H) or the positive mode loss of GlcNAc_1_P_1_Man_2_ (607 Da) are indicative of the HexNAcPMan modification; MS/MS of the product of HF treatment shows the underlying Man_7_GlcNAc_2_ isomer. **(J-L)** MALDI-TOF MS/MS of two glycans modified with unmodified phosphate and the α-mannosidase product. **(M)** MALDI-TOF MS/MS of a core fucosylated structure in the same fraction modified with methylphosphate. Further examples of phosphoester-modified glycans are shown in Supplementary Figures 3 and 4.

In both species analysed*,* the (methyl)phosphorylated N-glycans elute early on the RP-Amide column and vary from Hex_4–9_HexNAc_2_PMe_1–3_ and Hex_4–9_HexNAc_2–3_P_1_. As previously observed for *D. discoideum* [28], the phosphorylated structures were detected in positive and negative ion modes and were sensitive to HF treatments (Figs. 3 *and* 4 and ***Supplementary Figures 3 and 4***); when subject to jack bean α-mannosidase treatment alone, the Hex_7_HexNAc_2_-based structures “lost” up to three mannoses from the A and B arms (Figs. 3***D and*** 4***E***).

**Fig. 4:**
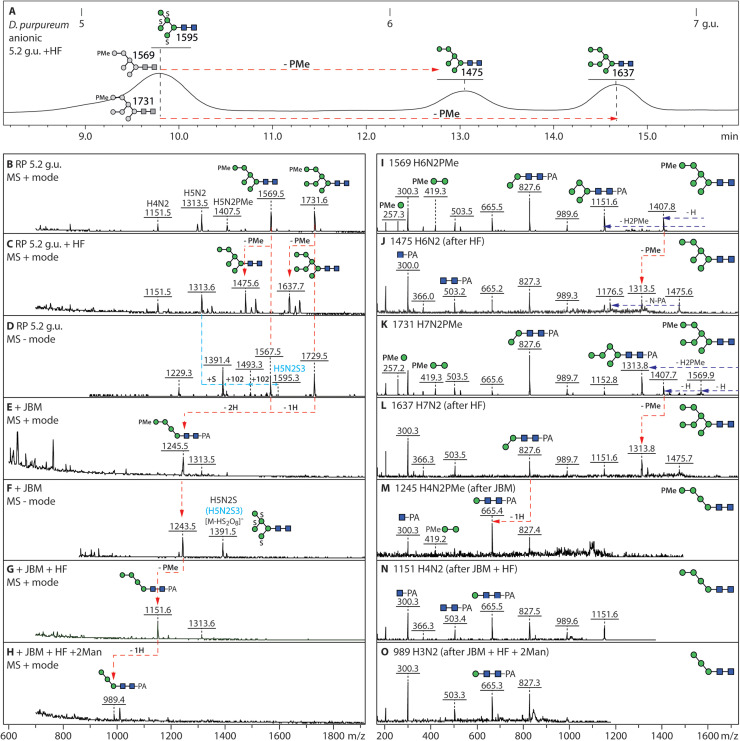
**Combined enzymatic, chemical and RP HPLC approach for definition of the methylphosphate modification. (A-D)** The 5.2. g.u. RP HPLC fraction of the *D. purpureum* anionic N-glycan pool contained two glycans with single methylphosphate modifications, which were removed by HF treatment resulting in a shift in retention time and mass, thereby definining the underlying oligomannosidic isomers. **(E-H)** In parallel, jack bean mannosidase digestion resulted in a major Man_4_GlcNAc_2_PMe, indicating that the C-arm was blocked by the methylphosphate moiety, which was then removed by subsequent HF treatment; finally, the dephosphorylated glycan was sensitive to α1,2-mannosidase, resulting in an α1,6-mannosidase-sensitive product of *m/z* 989. Note that the co-eluting *m/z* 1595 glycan (D) is insensitive to α-mannosidase digestion due to the three sulphate residues, which are labile during mass spectrometry, but resistant to HF. **(I-O)** Positive mode MALDI-TOF MS of the major methylphosphorylated glycans in the 5.2. g.u. fraction and their digestion products showing shifts in the pattern of B and Y-ions. Further examples of methylphosphorylated glycans are shown in Supplementary Figures 3, 4 and 5.

We assume that the primary location of these terminal modifications is on the C arm with either phosphate, methylphosphate or GlcNAc-1-phosphate blocking the action of the mannosidase (Fig. 3***D and*** 4***E/F***) as glycans of *m/z* 1229 Hex_4_HexNAc_2_P, *m/z* 1243 Hex_4_HexNAc_2_PMe and *m/z* 1432 Hex_4_HexNAc_3_P (as [M-H]^-^) are the major products. Subsequent treatment with HF, combined with RP HPLC, confirmed the co-elution of the product *m/z* 1151 (Hex_4_GlcNAc_2_) with a linear Man_4_GlcNAc_2_ isomer (Fig. 3***E/F***), which was also susceptible to α1,2-mannosidase (Fig. 4***G/H***) and α1,6-mannosidase, compatible with the phosphorylation of the C arm. Whereas glycans carrying unmodified phosphate were also sensitive to shrimp alkaline phosphatase (***Supplementary Figure 3***), all phospho-epitopes, i.e., phosphate, methylated phosphate and GlcNAc-1-phosphate, were removed by HF (Figs. 3 ***and*** 4 and ***Supplementary Figures 3 and 4***). In the case of direct HF treatment of the Hex_6–8_GlcNAc_2_(PMe)_1–3_ structures, a shift from early to late elution for the resulting “neutral” products was observed (Fig. 4***A*** and ***Supplementary Figures 3A and 4A***). The phospho-modifications could also be distinguished by positive or negative mode MS/MS (Figs. 3 ***and*** 4***, Supplementary Figure 3F-K*** and ***Supplementary Figure 4E-G***), due to the distinct B-ions at, e.g., *m/z* 243/405 (Man_1–2_P), 257/419 (Man_1–2_PMe) and, in negative mode, 444 (Man_1_PGlcNAc). Indeed, both species express glycans containing this GlcNAc-1-phosphate modification of the C-antennal terminal mannose residue (Fig. 3***G/H***), which is an intermediate during Man-6-phosphate biosynthesis in mammals and in *D. discoideum* [[51], [52], [53]]*.* In the case of glycans with multiple phosphoesters, generally both the A and C arms were modified resulting in key Y fragments of Hex_3_HexNAc_2_PMe-PA (*m/z* 1081/1083) and Hex_3_HexNAc_2_P-PA (*m/z* 1067/1069; ***Supplementary Figures 3F/H/I and 4E/F***), which upon HF treatment resulted in a *m/z* 989 fragment (***Supplementary Figures 3*** ***J and 4*** ***G***). It is noteworthy, that older NMR and MS data also showed that an intersected form of Man8A from *D. discoideum* carried methylphosphate on the A and C arms [54]. Indeed in both analysed species*,* similar N-glycan structures were observed (Hex_4–9_HexNAc_2_PMe_1–3_) with Man8A as the major underlying isomer. However, *D. giganteum* also contained fucosylated methylphosphorylated structures (Hex_6–7_HexNAc_2_Fuc_1–3_PMe_1_; ***Supplementary Figure 5***).

### 3.3 Unexpected neutral N-glycan epitopes with β 1,4 N-acetylglucosamine and galactose

**Fig. 5:**
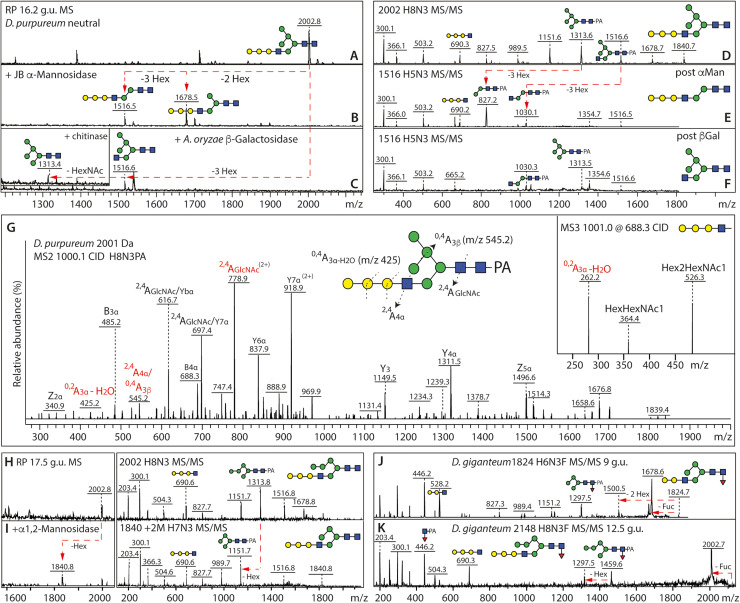
**MALDI TOF MS and LC-MS^n^ analysis of the galactose modified N-glycan in *D. purpureum* and *D. giganteum*.** Analysis of two isomeric N-glycan structures, separated by RP HPLC of the *D. purpureum* neutral N-glycan pool at 16.2 and 17.5 g.u (*m/z* 2002; Hex_8_HexNAc_3_) in positive ion mode with MALDI TOF MS/MS before and after exoglycosidase treatment. **(A-F)** Jack bean α-mannosidase or β1,4-specific galactosidase and subsequent chitinase (β1,3/4-hexosaminidase; see inset) treatment of the major isomer at 16.2 g.u. resulted in products of *m/z* 1516 and 1313 and shifts in the MS/MS spectra, as indicated with red arrows, indicative of a trigalactose motif on the lower arm, which correlated with the *m/z* 690 B-fragment; this glycan was insensitive to α1,2-mannosidase treatment (not shown). **(G)** Negative mode LC-MS^n^ of the major *D. purpureum* trigalactosylated Hex_8_HexNAc_3_ glycan shows antennal ^0.2^A-Gal and ^2,4^A-GlcNAc fragments compatible with a β1,4-linkage of the galactose residues. **(H, I)** The later eluting *m/z* 2002 isomer (17.5 g.u.) otherwise showing the same key *m/z* 690 and 1313 fragments, lost one hexose residue after α1,2-mannosidase treatment, indicative of a different underlying Man_5_GlcNAc_2_ backbone. **(J, K)** The galactosylated epitopes were also identified on some glycans from *D. giganteum*, whereby these structures were generally core α1,3-fucosylated as defined by the *m/z* 446 Y1-fragment ions, while *m/z* 528 and 690 B-fragments correlated with di- or trigalactose motifs on the “lower” A arm. Properties of the glycans with underlying non-reducing terminal GlcNAc are shown in Supplementary Figure 6 and further MS/MS of isomers with mono- up to penta-galactosylated epitopes are presented in Supplementary Figure 7.

In *D. purpureum*, it was apparent that many glycans possessed a modified lower arm. The first clue was that Hex_3–6_HexNAc_3_ glycans eluted rather late (2 g.u. later) as compared to those of the same composition in insect cells. Trials with jack bean hexosaminidase with subsequent HPLC suggested that the underlying structures were typical paucimannosidic N-glycans; thus, the late elution was proposed to be due to an unexpected GlcNAc linkage as compared to animal hybrid glycans, i.e., not β1,2-linked (***Supplementary Figure 6***). Larger glycans with a third HexNAc were hexosaminidase-resistant due to capping with a linear galactosylated motif, which was sensitive to β1,4-specific galactosidase digestion; together with data showing α-mannosidase sensitivity and removal of the third HexNAc by a β1,3/4-specific chitinase, but not a β1,2-specific hexosaminidase, the fragmentation pattern upon MALDI-TOF MS/MS and LC-MS^n^ was concluded to indicate the occurrence of a hybrid structure with a galactosylated β1,4-*N*-acetylglucosamine exclusively on the lower α1,3 mannose arm (Fig. 5***A-G***). As for some isomers of Hex_5–6_HexNAc_2_ mentioned above (***Supplementary Figure 2A-J***), there were also hybrid galactosylated structures predicted to contain a middle 'B' arm α1,2-linked mannose; in the case of the later-eluting *m/z* 2002 isomer, sensitivity towards α1,2-mannosidase was proven (Fig. 5***H/I***). On this basis and due to variations in fragmentation, a range of structures can be proposed, with up to five galactose residues in series as judged by the B ions ranging from *m/z* 528 up to 1014 (Hex_2–5_HexNAc_1_) complementary to the Y ions of *m/z* 989 up to 1475 (Hex_3–6_HexNAc_2_-PA; ***Supplementary Figure 7***). The galactosylated epitopes were also, to a lower extent, identified in *D. giganteum* and displayed similar relatively intense B-fragment ions at *m/z* 528 and 690 as found in *D. purpureum*, but these structures are often core α1,3-fucosylated as judged by the series of complementary Y ions at *m/z* 1297 and 1459 as well as the Y1 *m/z* 446 GlcNAc_1_Fuc_1_-PA fragment (Fig. 5***J/K***).

Some earlier-eluting isomeric glycans (Hex_5–8_HexNAc_3_) found in *D. purpureum* were concluded to possess an intersecting GlcNAc as in *D. discoideum* as also judged by their different MS/MS fragmentation pattern; e.g., there are five isomers of *m/z* 1516 containing either an intersecting motif, an unsubstituted 'lower' arm GlcNAc or a galactosylated motif (7.7, 8.8, 9.8, 10.5 and 12.5 g.u.; Fig. 1***A*** and ***Supplementary Figure 7 E and F***). Furthermore, there were up to seven isomers of *m/z* 1678, including an intersected form and various galactosylated forms with varying numbers of mannose and galactose residues (***Supplementary Figure 7 G-J***). No glycan contained both a 'lower' GlcNAc and an intersecting GlcNAc. Furthermore, in contrast to the cellular stage of *D. discoideum* [15], there were no bisected N-glycans detected in either *D. giganteum* or *D. purpureum*.

### 3.4 Multiple terminal fucosylated variants decorate mannosylated D. giganteum N-glycans

The overall neutral RP-HPLC chromatogram for *D. giganteum* shows a bias to early retention times; in combination with the frequent monofucosylated compositions, this is an indication for a high degree of core α1,3-fucosylation, which was less obvious in *D. purpureum*, which also has far more late-eluting galactosylated glycans. The Y ion fragment at *m/z* 446 was also indicative of fucosylation of the *N*-acetylglucosamine at the reducing end; sensitivity to hydrofluoric acid treatment and shift in elution verified the core α1,3-fucose modifications of different Man_5_GlcNAc_2_ isomers (***Supplementary Figure 8***). Further core modifications of the N-glycans such as mannosylation or xylosylation [55,56], galactosylation or methylation of fucose or multiple fucosylation [57,58] were not observed.

**Fig. 6:**
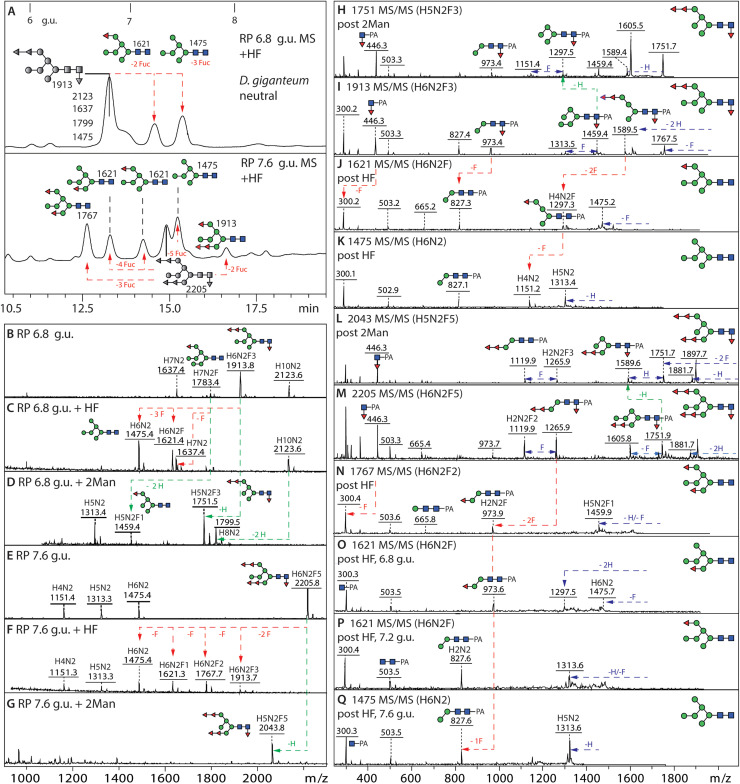
**RP-HPLC and MSMS analysis of N-glycans from *D. giganteum* modified with both core and terminal fucose. (A)** RP-Amide chromatograms of the two hydrofluoric acid (HF) treated 6.8 and 7.8 g.u. fractions of the neutral *D. giganteum* N-glycan pool showing shifts in retention time due to serial losses of fucose, revealing the underlying Man_6_GlcNAc_2_ structure. **(B-G)** MALDI-TOF MS of these fractions shows that the fucose residues, core linked to the reducing terminal *N*-acetylglucosamine residue and/or terminally linked to mannose were sensitive to HF treatment (red arrows), while specific α1,2-mannosidase treatment resulted in loss of one or two hexose residues (green arrows). **(H-Q)** Positive mode MALDI TOF MS/MS of the untreated, HF-treated and mannosidase-treated glycans highlights changes in the fragmentation patterns due to serial removal of fucose or removal of mannose. While the Y3-fragment at *m/z* 1265 (Hex_2_HexNAc_2_Fuc_3_-PA) is indicative of a fucosylated A arm, a fragment at *m/z* 973 (Hex_2_HexNAc_2_Fuc_1_-PA) can be either core or antennally fucosylated; a Y1 fragment at *m/z* 446 shows core fucosylation (see also Supplementary Figure 8). The final defucosylation products have Y3 fragments at *m/z* 827 (Hex_2_HexNAc_2_-PA). Note that the core α1,3-fucose was more sensitive than the antennal ones to HF treatment; for further multi-fucosylated isomers, refer to Supplementary Figure 9. Furthermore, the antennal fucose residues, but not the core α1,3-fucose, were partially released by bovine α-fucosidase, while all fucose residues were resistant to microbial α1,2-fucosidase and almond α1,3/4-fucosidase (data not shown).

Unexpected were the compositions indicative of multiple antennal fucose residues in *D. giganteum*, whereby the fucose rich fractions eluted on the RP-Amide between 5 and 17 g.u. and displayed a high degree of isomeric variation (Fig. 2). Two to six terminal fucose residues could be identified on oligomannosidic structures, whereby one or two fucose substitutions of any of the three mannose branches were determined, as shown by the sequential losses of fucose upon MS/MS and corroborative resistance or sensitivity to α-mannosidase treatment; some of these glycans were also core α1,3-fucosylated. Two typical structures (tri- or pentafucosylated) eluting at 6.8 and 7.6 g.u. were subject to HF treatment resulting in the same Hex_6_HexNAc_2_ mannosyl structure with a long middle arm (Fig. 6). HF treatment resulted in loss of both the core and anntenal fucose residues causing a shift of the RP-amide retention time and MS/MS fragmentation, while bovine kidney α-fucosidase partially removed the antennal fucose residues. Variants of further multiply fucosylated isomeric structures were assigned by B-ions such as *m/z* 1119 and 1265 (Hex_2_GlcNAc_2_Fuc_2–3_-PA; ***Supplementary Figure 9***). One trifucosylated glycan of *m/z* 2169 (Hex_7_HexNAc_2_Fuc_3_PMe_1_) was also found in the *D. giganteum* anionic fraction; it is presumed to be substituted with a methylphosphate on the α1,2-mannosylated C arm, two fucoses on the α 1,3 mannose arm and a core α1,3-fucose as judged by the Y fragment ions at *m/z* 446, 1119 and 1265 (***Supplementary Figure 5C***).

### 3.5 Sulphation of Dictyostelium N-glycans

Mannose-6-sulphate has been previously proposed as a modification of glycans from *D. discoideum* and indeed a range of sulphated and mixed sulphated/phosphorylated glycans have been detected in this species [16,34,59]. Sulphate differs from isobaric phosphate in terms of its ionisation in MALDI-TOF MS (primarily detectable in negative mode only), its resistance to hydrofluoric acid and, in a previous study, on the basis of highly exact mass spectrometry coupled to Fourier transform ion cyclotron resonance [28]. Based on these properties, we show that sulphation occurs in both *D. giganteum* and *D. purpureum.* Of the respectively 70 or 100 defined anionic N-glycans in these species, over half are proposed to be sulphated (***Supplementary*** ***Table 1***).

**Fig. 7:**
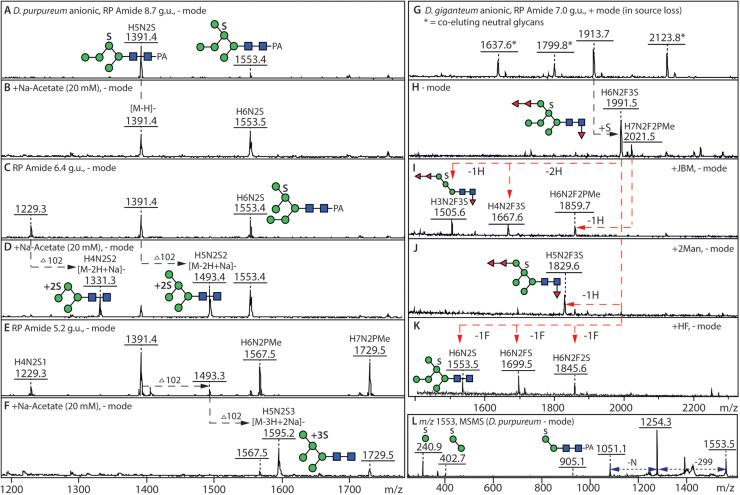
**Multiple sulphation in *D. purpureum* and fucosylated sulphated structures in *D. giganteum*. (A-F)** Negative mode MALDI TOF MS of single, double and triple sulphated mannosylated N-glycans of the anionic pool of *D. purpureum* in the three fractions (5.2 g.u., 6.4 g.u. and 8.7 g.u.) were selected for a comparison of the detection of multiple sulphation in negative ion mode; each additional sulphate residue results in an earlier RP-amide elution time. Addition of 20 mM sodium acetate to the 6-aza-2-thiothymine matrix facilitated detection of sodium adducts of di- and tri-sulphated glycans in negative ion mode. **(G-K)** The fucosylated sulphated structures in *D. giganteum* are more abundant and the position of the sulphate residues and the linkage of the fucose residues could be confirmed by a combination of jack bean α-mannosidase, α1,2-mannosidase and hydrofluoric acid treatments. One glycan structure from the anionic pool eluting at 7 g.u. was sensitive to jack bean mannosidase with a loss of up to three hexoses (from *m/z* 1991 Hex_6_HexNAc_2_Fuc_3_S to *m/z* 1505 Hex_3_HexNAc_2_Fuc_3_S) or sensitive to α1,2-mannosidase resulting in a product of *m/z* 1829 Hex_5_HexNAc_2_Fuc_3_S. HF treatment of the same glycan resulted in the loss of three fucose residues with a product of Hex_6_HexNAc_2_S indicating the HF-resistant sulphate is linked to mannose as in *D. purpureum* and *D. discoideum*, but not to fucose as in insects. **(L)** MALDI-TOF MS/MS of a sulphated N-glycan; the *m/z* 905 fragment as well as the pattern of α-mannosidase digestion indicates that the sulphate is probably on the core α1,6-mannose. For further examples of MS of sulphated glycans, refer to Supplementary Figure 10.

The sulphated glycans in *D. purpureum* (Fig. 1***B***) were primarily oligomannosidic structures, which were only partially sensitive to mannosidase treatment (Fig. 7 ***and Supplementary Figure 10***). The sulphated glycans in this species could also be modified with methylphosphate, whereby one Hex_6_HexNAc_2_ structure was concluded to contain one methylphosphate and four sulphate residues. Relatively few sulphated glycans in this species were also fucosylated and these were, like the neutral glycome, only core fucosylated. In contrast to *D. giganteum*, intersected glycans were found also in sulphated form. Due to the pattern of mannosidase resistance of these glycans and the otherwise trisubstitution of the upper arm α1,6-mannose residue in the intersected forms (i.e., an α1,3-mannose, an α1,6-mannose and the intersecting β1,4-GlcNAc), it can be surmised that the sulphate is 2-linked (***Supplementary Figure 10E***). No exact positions can be concluded for the more highly sulphated glycans (Man_4–8_GlcNAc_2_S_2–3_; ***Supplementary Figure 10*)**. Some of the sulphated glycans in this species were glucosylated, but none carried the galactosylated lower arm of the type found in the neutral glycomes.

As compared to *D. purpureum*, there were far fewer multiply-sulphated structures detected in *D. giganteum*, whereby the sulphation was present on a range of fucosylated and oligomannosidic backbone structures (Fig. 2***B***), generally with one sulphate on the upper arm α1,6-mannose residue (Fig. 7). The fucose residues were sensitive to HF treatment, while the sulphate was resistant. Based on this position, verified by resistance to mannosidase digestion, it can be presumed that the sulphate is either 2- or 4-linked to the otherwise 3,6-substituted mannose. While mannose-6-sulphate is known also from a lobster [60], 2- or 4-sulphation of hexoses is known in other eukaryotes, e.g., on polysaccharides from sea urchins or seaweeds [[61], [62], [63]].

## 4 Discussion

Dictyostelid cells are surrounded by a rich glycocalyx, whose glycan composition changes with the life stages [11,15,16]. In the comparative approach presented here, we define similarities and differences of the glycan epitopes in two Dictyostelid species, which share some motifs between each other and with the intensively studied species *D. discoideum*, but which also possess species-specific ones previously found not in other protists, fungi or invertebrates. While the N-glycomes of *D. purpureum* amoebae and slugs have been profiled by mass spectrometry [33], here we present a detailed analysis of N-glycans derived from its fruiting bodies, a stage also sampled for *D. giganteum*. Previous studies on *D. discoideum* have centered on the axenic AX3 strain and its lifecycle, but we also analysed the neutral N-glycans of the non-axenic NC4 [15]. These inter-species and inter-stage comparisons, indicate a certain degree of conservation of glycosylation motifs between Dictyostelid species, but also show diversities in terms of other epitopes.

**Fig. 8:**
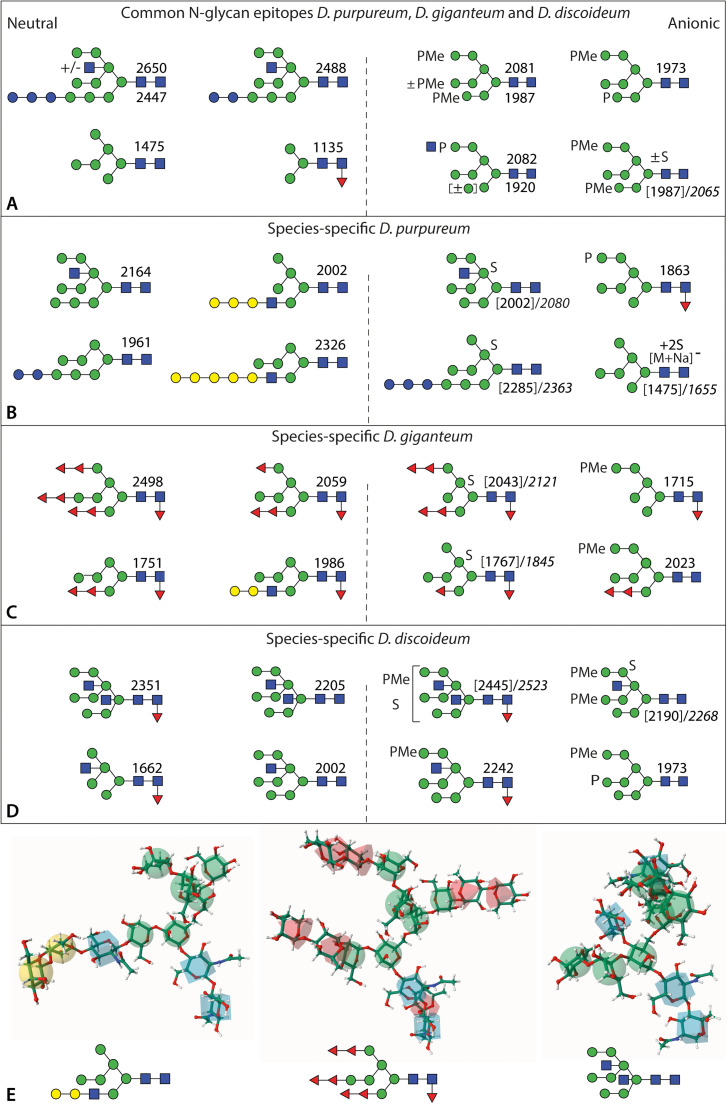
**Summary and comparison of the common and species-specific N-glycan epitopes in *Dictyostelium* species.** The depicted structures are exemplary neutral and anionic N-glycans common in all three *Dictyostelium* species **(A)**, as well species-specific to *D. purpureum***(B)**, *D. giganteum***(C)** and *D. discoideum***(D)**. Typical examples of neutral and anionic structures identified in all three species are modified with core α1,3-fucose; terminal glucose modification of the A arm (Glc_3_Man_9_GlcNAc_2_), methylphosphate and GlcNAc-1-phosphate (as biosynthetic intermediate) on a terminal α1,2-mannose and intersecting β1,4-*N*-acetylglucosamine are also features of all three. The linear galactosylated A arm modification linked to β-*N*-acetylglucosamine is specific to *D. purpureum*, while antennal fucosylation is a feature of *D. giganteum*, whereby the pattern of HF and fucosidase sensitivity suggest α1,2/3/4-linkages*.* In *D. discoideum,* intersecting and bisecting N-glycans modified core α1,3-fucose are specific to this species. Methylphosphorylation and sulphation occurs in all three species; however, the sulphate positions and linkages in *D. purpureum* and *D. giganteum* are not subterminal as in *D. discoideum*, but are probably on core α1,6-mannose residues. **(E)** 3D models of three typical species-specific N-glycans, prepared using the Glycam server [75], whereby α1,3-linkages were assumed for all fucose residues; the intersecting GlcNAc residue results in a 'bulky' conformation of the B and C arms as compared to structures galactosylated on the A arm or fucosylated on all three arms.

The vast majority of glycomic information on Dictyostelids is on *D. discoideum*, whereby a large number of classical studies by Freeze, Kornfeld, Henderson and West [12,24,26,54,64,65], complemented by our off-line LC-MS data [28,29,34], have shown a unique repertoire of glyco-motifs, including bisecting and intersecting β1,4-linked *N*-acetylglucosamine, core α1,3-fucosylation, sulphation of sub-terminal mannose residues and methylphosphorylation of terminal mannose residues. The comparative glycoprofiling study by West extended our knowledge to other related species, including *D. purpureum*. Methylphosphorylation and sulphation are common features amongst the five species examined in their study: only *D. discoideum* had glycans with four GlcNAc residues (i.e., the bisecting, intersecting and chitobiose residues; see Fig. 8 for a summary), while a maximum of three HexNAc residues per glycan was defined for other social amoebae, specifically *D. fasciculatum, Polysphondylium pallidum* and *Actyostelium subglobosum* [33].

Indeed, in *D. giganteum* and *D. purpureum*, we can now confirm maximally three HexNAc residues per glycan which results from a lack of bisecting GlcNAc; however, in *D. purpureum* the third GlcNAc is either the 'intersecting' one substituting the 'upper' core α1,6-mannose or the 'antennal' one on the 'lower' core α1,3-mannose, both β1,4-linked (see Fig. 8). In glycogenomic terms, this may correlate with the presence of homologues of the mammalian bisecting *N*-acetylglucosaminyltransferase III (Mgat3; CAZy GT17) in the genome of *D. discoideum*, but which are absent from *D. purpureum* [*6*]; this may mean that the intersecting and lower arm β1,4-*N*-acetylglucosaminyltransferases in *D. purpureum* are members of other glycosyltransferase families. The proposed β1,4-linkage for the antennal A-arm GlcNAc as well as the lack of relevant homologues indicate that the GlcNAc-TI pathway (Mgat1; CAZy GT13), known for multicellular organisms, is absent from these protists; also, no N-glycan contained both an A-arm GlcNAc and an intersecting GlcNAc. On the other hand, both *D. discoideum* and *D. purpureum* possess multiple GT10 α1,3-fucosyltransferase homologues and one GlcNAc-1-phosphotransferase (*gpt*) homologue compatible with the occurrence of core α1,3-fucose and mannose-6-phosphate epitopes in all three species [6], whereby only the biochemical function of the *gpt* gene has been specifically proven [53]. Core α1,3-fucosylation is widespread in plants and invertebrates, correlating with expression of anti-horseradish peroxidase epitopes [66], while mannose-6-phosphate is only known from amoebae and vertebrates. While the phosphates are often methylated, no other methylated residue was detected in this study, unlike the methylmannose previously found in *P. pallidum* [33]. The presence of core α1,6-fucose as found in *Acanthamoeba* strains [55] and in animals has been claimed in only one *D. discoideum* glycoprotein [67], but we have never proven this in our own studies.

The other significant difference between the three species is in the occurrence of the multiple fucose residues on *D. giganteum* and the elongated galactose antenna in both *D. giganteum* and *D. purpureum*, which is especially pronounced in the latter, whereby antennal fucosylation and antennal galactosylation are mutually exclusive. These two features are absent from *D. discoideum*, but the genomic basis is not clear, especially as there is no gene sequence information on *D. giganteum.* Galβ1,4 Gal motifs are known from *C. elegans*, as a modification of core α1,6-fucose [68], and in fish or birds, as a modification of antennal GlcNAc residues [69,70], which indeed is closer to the context observed here. Fucosylated fucose epitopes are known from schistosomes as Fucα1,2Fucα1,3HexNAc motifs [71]; however, fucosylation of mannose residues has been reported in fungi: specifically the modification of α1,6-mannose residues by α1,6-fucose [72]. Due to the pattern of HF sensitivity shown here, we assume that the fucose residues linked to mannose are either α1,2- or α1,3/4-linked, as fucose α1,2-linked is partially susceptible, α1,3/4-linked fully susceptible and α1,6-linked resistant to this treatment [73]; the partial sensitivity of the glycans to bovine kidney α-fucosidase, which prefers core α1,6-fucose, but also removes antennal fucose, including oligofucose motifs [68,71], indicates the occurrence of the latter without defining the actual linkages. On the other hand, the core fucose residue is certainly α1,3-linked in all *Dictyostelium* species analysed in our hands. The amounts of fruiting bodies isolated preclude NMR or GC/MS studies to absolutely define all linkages of individual structures; nevertheless, the extent of fucosylation in *D. giganteum* distinguishes it from other social amoebae.

More subtle are the variations in sulphation: while mannose-6-sulphation of subterminal mannose residues has been established in *D. discoideum* [12,59], the main position in *D. giganteum* and *D. purpureum* is rather on the 'upper' core α1,6-mannose residue. In the case of a sulphated intersected glycan, the result is a tetra-substituted α1,6-mannose residue modified with 3- and 6-linked mannose, the 4-linked intersecting GlcNAc and, by process of elimination, a 2-linked sulphate. In the case of di-, tri- and tetrasulphated glycans, it is an open question as to whether 6-sulphation also occurs. Such multiply-sulphated structures as well as mixed sulphated/methylphosphorylated ones were not previously described in *D. purpureum*, but sulphate residues tend to be labile and so are more difficult to detect than methylphosphorylated ones. In *D. giganteum*, many sulphated structures were found to be fucosylated, while *D. purpureum* also had some glucosylated glycans carrying sulphate, but galactosylated glycans were found in neither of the anionic glycan pools. This suggests that some modifications are mutually exclusive, either due to specific 'signals' or stereochemical restraint.

All the N-glycans in the Dictyostelium species probably originate from the usual eukaryotic dolichol-linked Glc_3_Man_9_GlcNAc_2_ precursor, which has also been specifically isolated from *D. discoideum* in the lipid-linked form [28,74]. As previously noted for *D. purpureum* cells and slugs [33], residual glucosylated N-glycans are found in both *D. giganteum* and *D. purpureum* fruiting bodies (especially in the latter), whereas such structures are common in *D. discoideum* only in the modA glucosidase II mutant [29]. Some of the glucosylated structures in *D. purpureum* are also carrying intersecting GlcNAc or sulphate, which may indicate that this species has a slightly lower degree of glucosidase activity in the endoplasmic reticulum. Overall, the *D. pupureum* N-glycome has a broad range of glycans based on Hex_3–12_HexNAc_2_, while in *D. giganteum* fucosylated glycans with Hex_2–6_HexNAc_2_ backbones are dominant. In contrast, the *D. discoideum* glycome is dominated by Man_9_GlcNAc_2_ and Man_8_GlcNAc_4_Fuc_1_ at the cellular stage, but truncated glycans in later stages [15] occur due to a high degree of Golgi mannosidase activity [26].

In methodological terms, the combination of pre-fractionation into neutral and anionic pools, followed by off-line LC/MS, has again showed its value in the isomeric/isobaric separation of N-glycans. The use of the fused core RP-amide HPLC column enables excellent separation, whereby phosphorylated and core α1,3-fucosylated glycans elute relatively earlier, while the middle B-arm α1,2-mannose and the antennal fucose and galactose modifications result in later elution. Monosulphation made little difference to retention time as compared to the parent structure, but multiple sulphate residues result in earlier elution.

## 5 Conclusion

The N-glycome analysis of the fruiting bodies from both species *D. purpureum* and *D. giganteum* resulted in a number of surprises. The mass spectrometric definition of the structures combined with chemical or enzymatic treatments was applied to neutral and anionic glycan pools enabling the assignment of approximately 170 structures per strain. The neutral N-glycans of *D. purpureum* are not trimmed as observed in *D. discoideum*; in contrast they display glucosylated and galactosylated modifications and less fucosylation which may indicate low mannosidase, glucosidase and fucosyltransferase activity. However, highly fucosylated N-glycan epitopes were observed in the neutral pool of *D. giganteum* with mannose-fucose and fucose-fucose linkages previously unknown in amoeba. In the anionic pools, phosphate modifications with and without methyl groups are common in all Dictyostelium species analysed here or previously. However, the position of sulphate linked to mannose differs in *D. giganteum* and *D. purpureum* as compared to *D. discoideum*. The N-glycan biosynthetic pathways of especially *D. giganteum* can unfortunately not be predicted due to a lack of genomic sequence data. However, our data on fruiting bodies of different species demonstrates that speciation in the Dictyostelids is accompanied by glycomic diversification.

## CRediT authorship contribution statement

**Alba Hykollari:** Writing – review & editing, Writing – original draft, Visualization, Investigation, Funding acquisition. **Daniel Malzl:** Formal analysis, Visualization. **Chunsheng Jin:** Investigation, Formal analysis. **Carina Eschenbach:** Investigation. **Kristína Kianičková:** Data curation. **Iain B.H. Wilson:** Writing – review & editing, Writing – original draft, Investigation. **Katharina Paschinger:** Writing – review & editing, Investigation, Funding acquisition, Formal analysis.

## Declaration of competing interest

The authors declare the following financial interests/personal relationships which may be considered as potential competing interests:

Katharina Paschinger reports financial support was provided by Austrian Science Fund. If there are other authors, they declare that they have no known competing financial interests or personal relationships that could have appeared to influence the work reported in this paper.

## Data Availability

Selected mzxml files are available on Glycopost, https://glycopost.glycosmos.org/entry/GPST000532.

## Appendix Supplementary materials

Image, application 1
