# Supplementary material for: New insights into the N-glycomes of Dictyostelium species
Source: BBA Adv. 2025 Jan 16;7:100142. doi: 10.1016/j.bbadva.2025.100142 (PMC11795075; doi:10.1016/j.bbadva.2025.100142)
Supplement: Supplementary file 1 [file mmc1.pdf]

NEW INSIGHTS INTO THE N-GLYCOMES OF *DICTYOSTELIUM* SPECIES

Alba Hykollari, Daniel Malzl, Chunsheng Jin (金春生), Carina Eschenbach,
Kristína Kianičková, Iain B. H. Wilson, Katharina Paschinger

Supplementary Information

Further information regarding the glycomic analyses

Definition of the level of the glycan structural analysis:

The goal was the N-glycomic analysis of the fruiting bodies of two different *Dictyostelium* species (*D. giganteum* and *D. purpureum*). Thereby, whole N-glycomes and individual glycan-containing HPLC fractions were subject to MALDI-TOF MS and MS/MS.

Search parameters and acceptance criteria:

a. **Peak lists:** As stated in the methods section: typically 1000-4000 shots were summed for MALDI-TOF MS and 5000-20000 for MS/MS. Spectra were processed with the manufacturer's software (Bruker Flexanalysis 3.3.80) using the SNAP algorithm with a signal/noise threshold of 6 for MS (unsmoothed) and 3 for MS/MS (four-times smoothed).

b. **Search engine, database and fixed modifications:** All glycan data were manually interpreted and no search engine or database was employed; the fixed modification is the 2-aminopyridine label at the reducing end ($GlcNAc_1$-PA fragments of *m/z* 300); negative mode fragments (e.g., *m/z* 241, 255 and 444) are indicative of phosphate/sulphate, methylphosphate and GlcNAc-1-phosphate modifications of mannose.

c. **Exclusion of known contaminants and threshold:** All glycan data were manually interpreted; only peaks with an MS/MS consistent with a pyridylaminated chitobiose core were included – the 'threshold' for inclusion was an interpretable MS/MS spectrum (at least in terms of composition).

d. **Enzyme specificity:** A description of the PNGase F and A release method is given in the methods section; this combination of enzymes should remove N-glycans from glycopeptides regardless of the presence of core α1,3-fucose on the reducing-terminal GlcNAc. Exoglycosidase digestions were performed on selected glycan fractions to determine linkages of some glycan motifs.

e. **Isobaric/isomeric assignments:** For isomeric species, differences in RP-HPLC elution, MS/MS and exoglycosidase sensitivity were used for the assignment (as described in the text). For isobaric structures (e.g., sulphate and phosphate) hydrofluoric acid treatment and enzymatic digest (shrimp alkaline phosphatase) were applied.

Glycan or glycoconjugate identification:

a. **Precursor charge and mass/charge (*m/z*):** All glycans detected were singly-charged. For the positive mode, the *m/z* values are for protonated forms. Depending on the glycan amount or preparation, the relative amounts of the H^+, Na^+ and K^+ adducts varied. Maximally one decimal place used for the *m/z* annotations consistent with the accuracy of MALDI-TOF MS; in the figures and due to space limitations, only one decimal place is presented. Previous data indicate an average +0.03 Da (+ 22 ppm) deviation between the measured and the calculated *m/z* values on the instrument used.

b. **MALDI-TOF MS settings (positive mode):** For Autoflex Speed: Ion Source 1 and 2 were 19.00 and 16.75 kV; Lens, 9.00 kV; Reflector 1 and 2, 21.05 and 9.65 kV; Pulsed Ion

Extraction, 160 ns; Matrix Suppression typically up to 700 Da; Detector Gain, typically 2163 V. For Rapiflex: reflector voltage, lens voltage, and gain of 20.8 kV, 11.6 kV, and 1909 V respectively.

c. **MALDI-TOF MS/MS settings (positive mode):** For Autoflex Speed: Ion Source 1 and 2 were 6.00 and 5.35 kV; Lens, 2.90 kV; Reflector 1 and 2, 27.00 and 11.75 kV; Lift 1 and 2, 19.00 and 4.00 kV; Pulsed Ion Extraction, 140 ns; Detector Gain, typically 2260 V when fragmenting; Laser Power Boost typically 50%; not in CID mode; PCIS typically 0.65%. For Rapiflex: reflector voltage, lift voltage, and gain of 23.8 kV, 19 kV, and 2171 V respectively; PCIS 0.3%.

d. **All assignments:** For the glycans present in each pool, see the RP-HPLC chromatograms annotated with structures shown according to the Standard Nomenclature for Glycans.

e. **Modifications observed:** Listed are the *m/z* values for glycans carrying a reducing terminal pyridylamine group as judged by presence of an *m/z* 300 $GlcNAc_1$-PA fragment. As the glycans are otherwise chemically unmodified, $\Delta m/z$ of 80, 94, 102, 146, 162 and 203 correspond to phosphate or sulphate (detected in negative mode), methylphosphate, sulphate in sodiated form, fucose (deoxyhexose), hexose, or *N*-acetylhexosamine. Pyridylamine is a reducing-terminal chemical modification of the N-glycans performed during the glycomic workflow.

f. **Number of assigned masses:** Glycan assignments were not just based on measured mass only, but on the basis of MS/MS corroborated by elution data.

g. **Spectra:** Representative annotated spectra (MS and MS/MS) defining structural elements are given in various figures. In total, over 2200 MS or MS/MS spectra reflecting the data for the approximately 145 defined structures are shown in the main text or supplement; 65 example MS and MS/MS are available as mzxml (Glycopost, GPST000532).

h. **Structural assignments:** As noted in the results section, the typical oligomannosidic structures are assigned based on elution time and fragmentation pattern; it is otherwise assumed that the glycans contain a tri-mannosyl core consistent with typical eukaryotic N-glycan biosynthesis and that there is processing by GlcNAc-transferases, sulphotransferases, *N*-acetylglucosamine-1-phosphotransferases and core α1,3-fucosyltransferases. The sulphated glycans are observed in negative mode as $[M-H]^-$ ions, but in-source loss results in the observation of $[M+H-80]^+$ ions in positive mode - a property contrasting with phosphorylated glycans observed in both modes.

Summary of glycosidases and phosphatases used in this study

PNGase	*Oryza sativa* PNGase Ar (recombinant, *Pichia*)
	Flavobacterium PNGase F (recombinant, *E. coli*)
Fucosidase	Bovine kidney α-fucosidase (Sigma)
Galactosidase	*Aspergillus oryzae* β-galactosidase (native)
Hexosaminidases	Jack bean β-*N*-acetylhexosaminidase (native)
	Streptomyces plicatus chitinase (recombinant, *E. coli*)
	Xanthomonas manihotis β1,2-*N*-acetylglucosaminidase (recombinant, *E. coli*)
Phosphatase	Shrimp alkaline phosphatase
Mannosidases	Jack bean α-mannosidase (recombinant, *Pichia*)
	Aspergillus α1,2-mannosidase (native)
	Xanthomonas manihotis α1,2/3-mannosidase (recombinant, *E. coli*)
	Xanthomonas manihotis α1,6-mannosidase (recombinant, *E. coli*)
	Bacteroides xylanisolvens endo-α-mannosidase (recombinant, *E. coli*)

Figure S1: Glycomic workflow employed in this study and MALDI TOF MS N-glycome spectra of complete neutral and anionic pools of *D. purpureum* and *D. giganteum* N-glycans. (A) Summary of the “Experimental Procedures” indicating serial digestion with PNGase F and PNGase Ar followed by solid-phase extraction and labelling steps. **(B)** Positive mode MALDI-TOF MS spectra of the neutral pools and negative mode MALDI TOF MS of the anionic pools after pyridylamination are annotated with the *m/z* values, respectively $[M+H]^+$ or $[M-H]^-$, and abbreviated compositions (F, fucose; H, hexose; N, *N*-acetylhexosamine; S, sulphate; P, Phosphate; Me methyl). The strong signals in the negative ion mode of the anionic-enriched pools indicate the presence of sulphated and phosphorylated N-glycans. **(C)** Comparison of the pyridylaminated neutral N-glycans of three Dictyostelid species analyzed at the very late developmental stage (fruiting bodies). The *D. discoideum* N-glycome contains all three types of modifications typical for the cellular stage; *i.e.* mannose trimmed species modified with core fucose and $Hex_8HexNAc_{2-4}$ structures modified with intersecting and bisecting GlcNAc (epitopes identification also described in previous studies). The N-glycomes of *D. purpureum* and *D. giganteum*, consist of common structures for fruiting bodies ($Hex_{2-6}HexNAc_2$) with some species-specific modifications, such as the highly fucosylated structures in *D. giganteum* and the long hexose modifications in *D. purpureum*. The A, B and C arms as well as intersecting GlcNAc are defined.

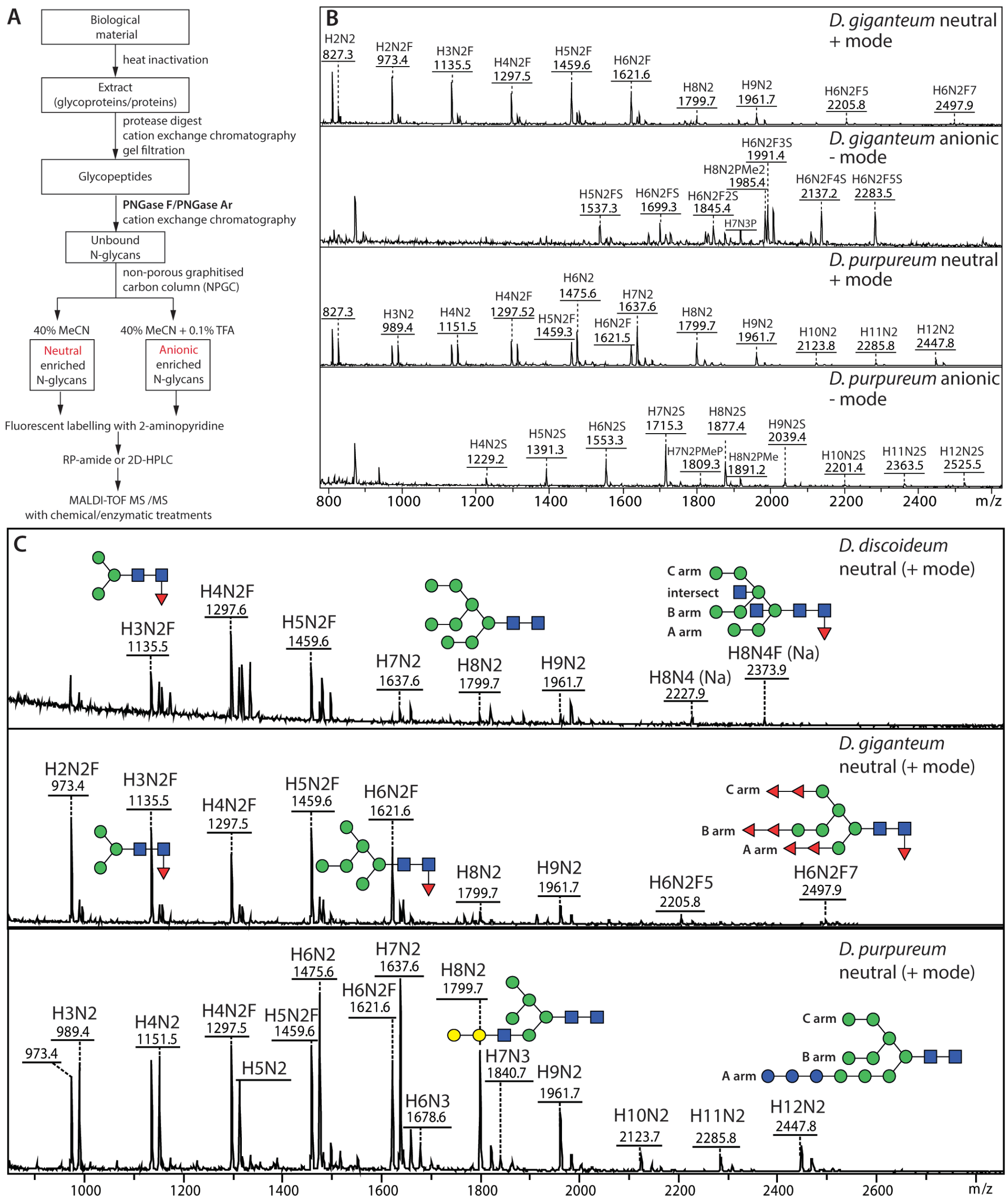

Figure S2: (A-J) Analysis of multiple Man$_5$GlcNAc$_2$ and Man$_6$GlcNAc$_2$ isomers separated on RP-amide. MALDI-TOF MS/MS of *m/z* 1313 and 1475 isomers from *D. purpureum* eluting at different glucose units (g.u.; the numbers in round brackets are those defined by Tomiya *et al.* on a C18 column), some of which were incubated with *Aspergillus* α1,2-mannosidase with removal of 0-2 hexose units; selected diagnostic Y fragments are annotated, whose relative intensities either do not shift (E) or do shift (G) upon α1,2-mannosidase depending on whether the A or B/C arms are extended (see spectra in red). An intense *m/z* 665 Y fragment is indicative for a complete lack of an A arm (C/H), while an intense *m/z* 827/989 indicates presence of an A arm with or without an α1,2-mannose. **(K-O) Analysis of unprocessed glucosylated neutral N-glycans from *D. purpureum*.** MALDI TOF MS of *m/z* 2447 (Hex$_{12}$HexNAc$_2$; 6.8 g.u.) and *m/z* 2489 (Hex$_{11}$HexNAc$_3$; 9.4 g.u.) before and after treatment with α1,2-mannosidase and *Bacteroides xylanisolvens* endo-α-mannosidase (EndoMan), whereby the glucosylated arm blocks the action of the α1,2-mannosidase, but is sensitive to the endomannosidase. **(P-T)** MALDI TOF MS/MS in positive ion mode of the substrates and products with changes in the key Y3-fragment ions as annotated. For the RP-amide HPLC chromatograms of pyridylaminated *D. purpureum* N-glycans refer to Figure 1 in the main text. Ten MS/MS of larger oligomannosidic glycans from *D. giganteum* are available as mzxml files accessible via Glycopost GPST00053.

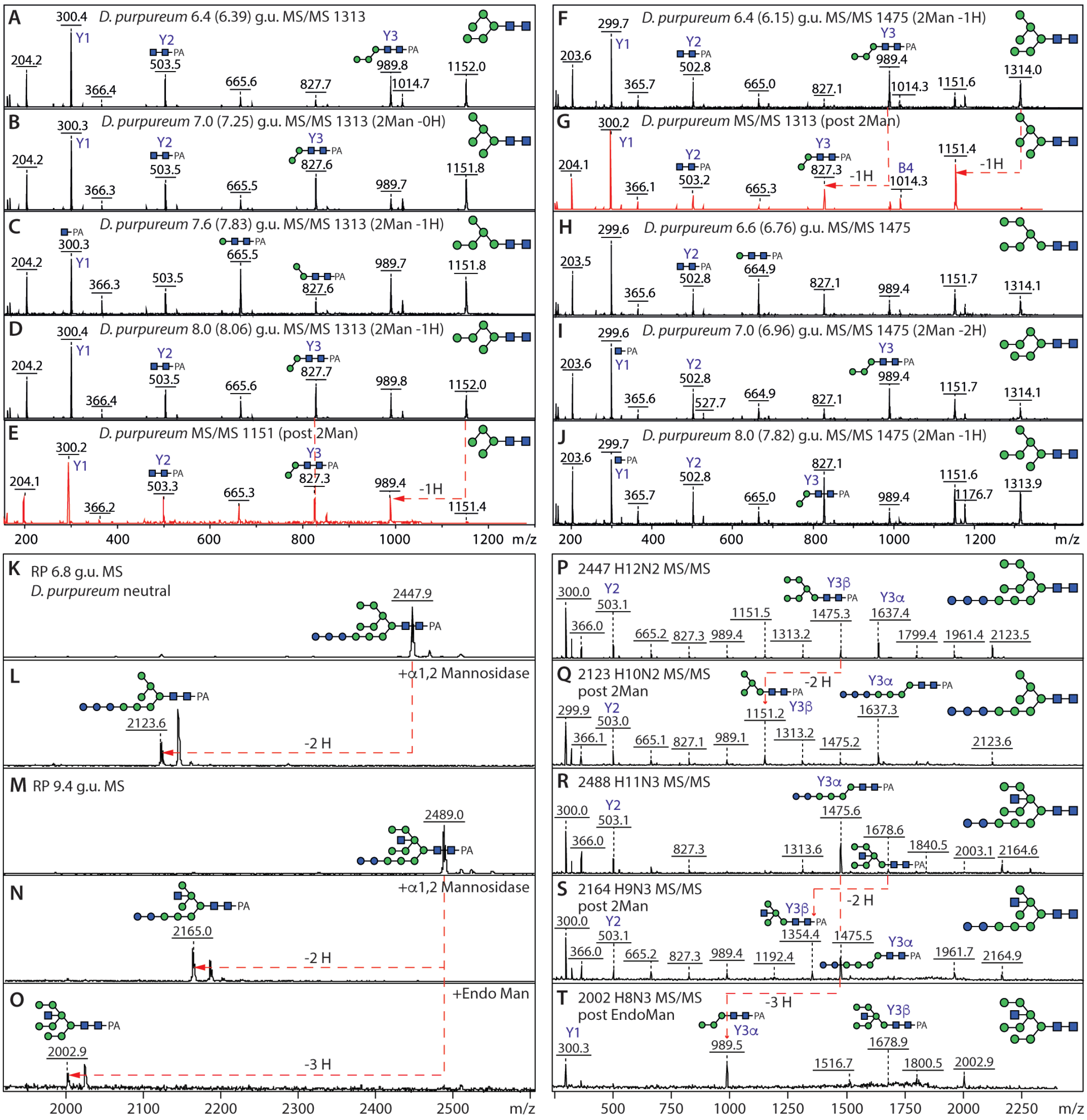

Figure S3: **Definition of a phosphorylated and methylphosphorylated anionic N-glycan in *D. purpureum***. **(A)** Chromatograms of one fraction from *D. purpureum* anionic N-glycan pool (2.2 g.u., see also Figure 1B) re-injected after either hydrofluoric acid (HF) or shrimp alkaline phosphatase (SAP) treatments. The collected fractions were analysed with MALDI TOF MS/MS and the N-glycans identified are annotated above the corresponding peaks. The four original N-glycans of *m/z* 1811, 1879, 1863, 2081 are depicted in grey. Loss of phosphate after SAP treatment is shown by red arrows, while loss of core α1,3-fucose (-146 Da), phosphate (-80 Da) and methylphosphate (-94 Da) due to HF treatment is indicated by blue arrows. **(B-C)** Positive and negative ion mode MALDI TOF MSMS of the fraction eluting at 2.2 g.u.; **(D and E)** Positive mode MALDI TOF MS after HF or SAP treatment. **(F-I)** MALDI TOF MS/MS analysis of *m/z* 1811, 1863,1879 and 2081 showing fragments at *m/z* 1069 or 1083 and 1407 indicative for the respective position of phosphate and methylphosphate. **(J-K)** MALDI TOF MS/MS of the *m/z* 1637 $Hex_7HexNAc_2$ product after HF treatment and *m/z* 1731 $Hex_7HexNAc_2PMe$ after SAP treatment showing the different B and Y-fragment ions; an MS/MS loss of 418 corresponds to Hex_2PMe. While phosphate, methylphosphate and core α1,3-fucose are all sensitive to HF, only the phosphate is removed by shrimp alkaline phosphatase, thus aiding definition of the *m/z* 1811 glycan as being modified with both phosphate and methylphosphate. The oligomannosidic products have the same elution positions and fragmentation patterns as the relevant oligomannosidic isomers in the neutral pool (see Figure 1A).

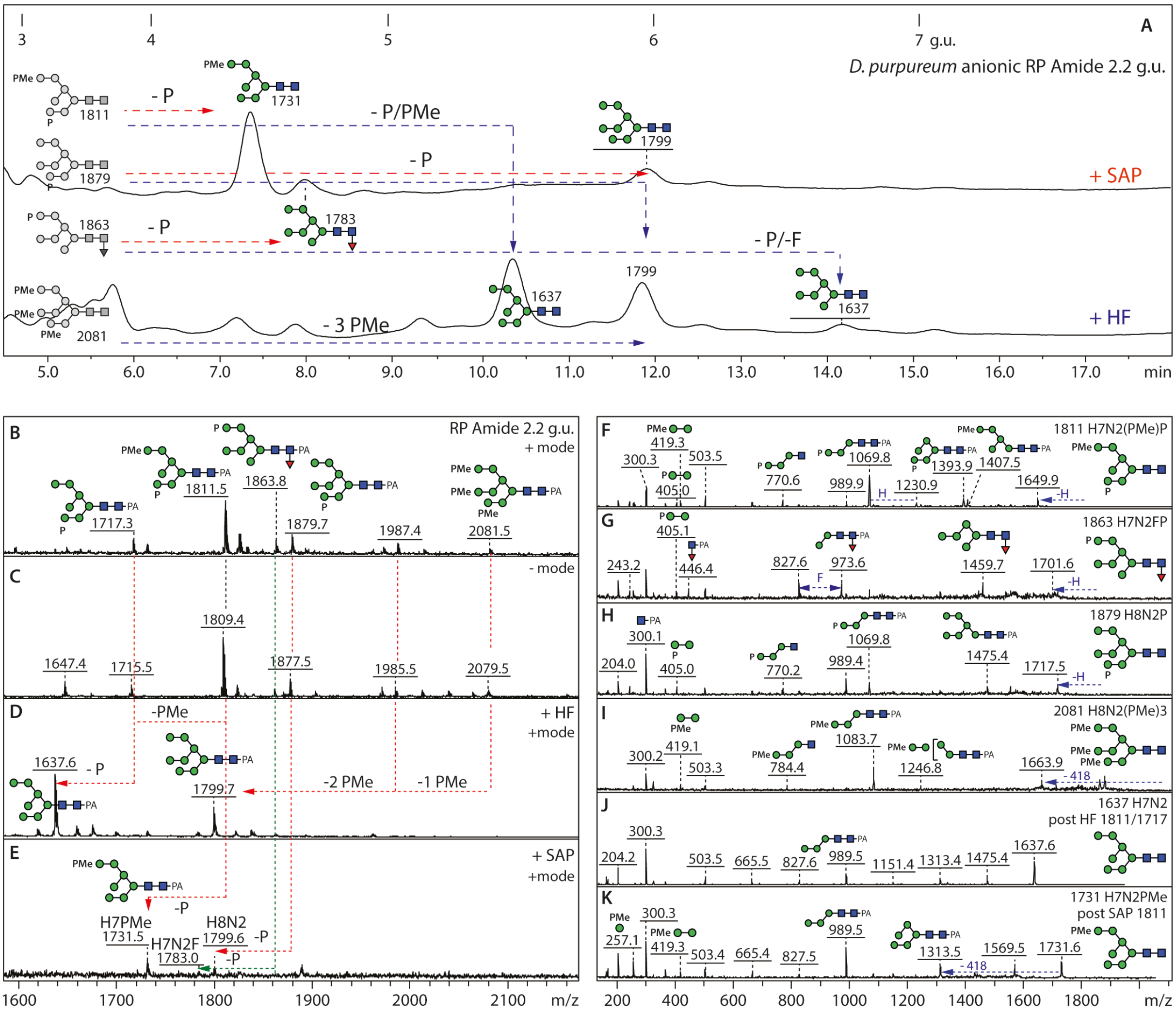

Figure S4: Methylphosphorylated anionic structures in *D. purpureum* can be analysed in positive and negative ion modes. (A) Two co-eluting (3 g.u.) anionic N-glycan structures of *m/z* 1825 and 1987 (in greyscale) were treated with hydrofluoric acid (HF) and analysed with MALDI TOF MS **(B-D)** before re-injection onto a RP column; the retention times were compared for co-elution with the neutral N-glycan pool. The HF treatment efficiently removed the methylphosphate modifications. **(E, F)** MALDI TOF MS/MS of *m/z* 1825/1823 in positive and negative ion modes; the $Hex_3HexNAc_2PMe$-PA Y "lower arm" fragments originating from loss of the upper arms were identified in both modi (*m/z* 1084/1082), whereas the B ions included those at *m/z* 741 and 903, i.e., representing the complete “upper” ($Hex_{4-5}PMe$) arm fragments; therefore, the methylphosphate residues are assumed to modify the A and C arms as in *D. discoideum* (Ref. 54). **(G)** After the HF treatment, the dephosphorylated structure of *m/z* 1637 was fragmented in positive ion mode, showing a key lower arm Y3 fragment ion at *m/z* 989, indicative of the A arm of the underlying isomeric structure. The second product of *m/z* 1799 eluted as expected for a Man8A isomer (ca. 6 g.u., as compared to 5.2 g.u. for Man8B).

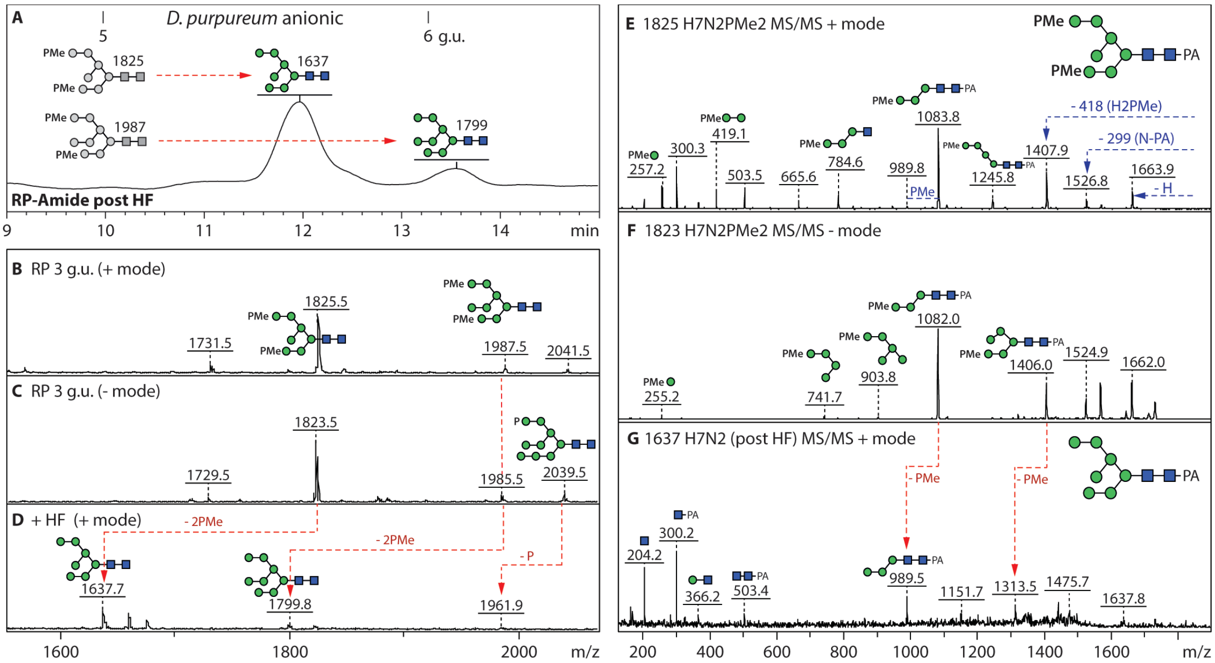

Figure S5: MALDI TOF MSMS examples of N-glycans modified with methylphosphate and fucose in *D. giganteum*. (A, C) Example of positive mode MS/MS of fucosylated and methylphosphorylated N-glycans ($Hex_7HexNAc_2Fuc_{1-3}PMe$; *m/z* 1877 and 2169) from the *D. giganteum* anionic pool eluting at 3.5 and 4.8 g.u. respectively. The *m/z* 446 and 649 fragments are indicative of the core fucose residue in both structures, whereas the Y3 fragments at *m/z* 1119 and 1265 show antennal fucosylation. **(B)** Treatment of the *m/z* 1877 N-glycan with α1,2-specific mannosidase (2Man) resulted in loss of one mannose yielding a $Hex_6HexNAc_2Fuc_1PMe$ product (*m/z* 1715) lacking one mannose residue of the "middle" B-arm, as indicated by a shift in the Y3 ion from *m/z* 1459 to *m/z* 1297 ($Hex_4HexNAc_2Fuc_1$). Furthermore, the *m/z* 1877 glycan was HF sensitive (loss of Fuc and PMe) and the *m/z* 2169 glycan also lost one hexose upon α1,2-mannosidase treatment (data not shown). The *m/z* 256 and *m/z* 419 $Hex_{1-2}PMe$ fragments were identified in all three structures. Assuming a greater lability of the 1,6-linkages carrying longer antennae and by comparison to *D. discoideum*, the methylphosphate is presumed to be on the C-arm, also as PMe modification of the A arm is ruled out by the dominant fucosylated Y3 *m/z* 973 or 1265 fragments. Refer to Figure 2 in the main text for the HPLC chromatograms of *D. giganteum* N-glycans.

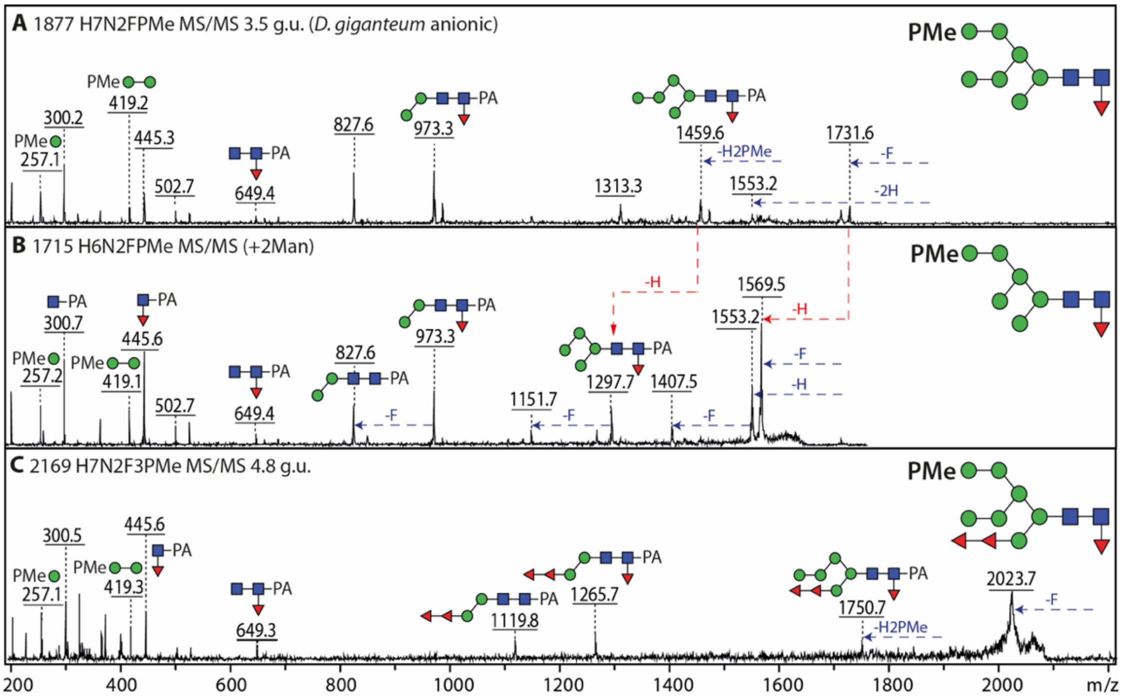

Figure S6: Neutral *D. purpureum* N-glycans modified with β1,4*N*-acetylglucosamine. (A) RP-HPLC of N-glycans eluting at 8.8 and 10.0 g.u. modified with terminal β1,4-*N*-acetylglucosamine before and after jack bean hexosaminidase treatment; as indicated, the *D. purpureum* structures elute later than the insect (honeybee) hybrid β1,2-*N*-acetylglucosamine isomers (black arrows). The hexosaminidase products elute 2 g.u. earlier than the original glycans (red arrows) and co-elute with known oligomannosidic glycans. **(B-F)** Positive ion mode MALDI TOF MS of the 8.8. g.u. fraction before and after hexosaminidase or mannosidase treatments; the resulting losses of hexoses or *N*-acetylhexosamine are highlighted in red. The *D. purpureum* β1,4-linked *N*-acetylglucosamine linked to the "lower" α1,3-mannose arm was sensitive to jack bean hexosaminidase and specific chitinase treatments, but insensitive to the *X. manihotis* β1,2-specific hexosaminidase; furthermore, jack bean mannosidase treatment resulted in loss of up to three hexose residues; the co-eluting glucosylated glycans were also partially mannosidase sensitive. The intersecting β1,4-*N*-acetylglucosamine identified in the $Hex_{10}HexNAc_2$ structure could not be released by hexosaminidase digestion (see also Refs. 29 and 34 for blocking of mannosidase action by the intersecting GlcNAc in a *D. discoideum*

mutant glycome). **(G-K)** Corresponding MS/MS spectra for the original and digested *m/z* 1516 and *m/z* 2123 glycans showing alterations in the pattern of Y fragment ions. **(L-O)** MALDI TOF MS of the *m/z* 1678 $Hex_6HexNAc_3$ N-glycan eluting at 10 g.u. before and after jack bean hexosaminidase (JBH), jack bean mannosidase (JBM) or *Aspergillus* α1,2-mannosidase (2Man) treatments. **(P-S)** Corresponding MS/MS spectra for the original and digested *m/z* 1678 glycan showing alterations in Y fragment ions.

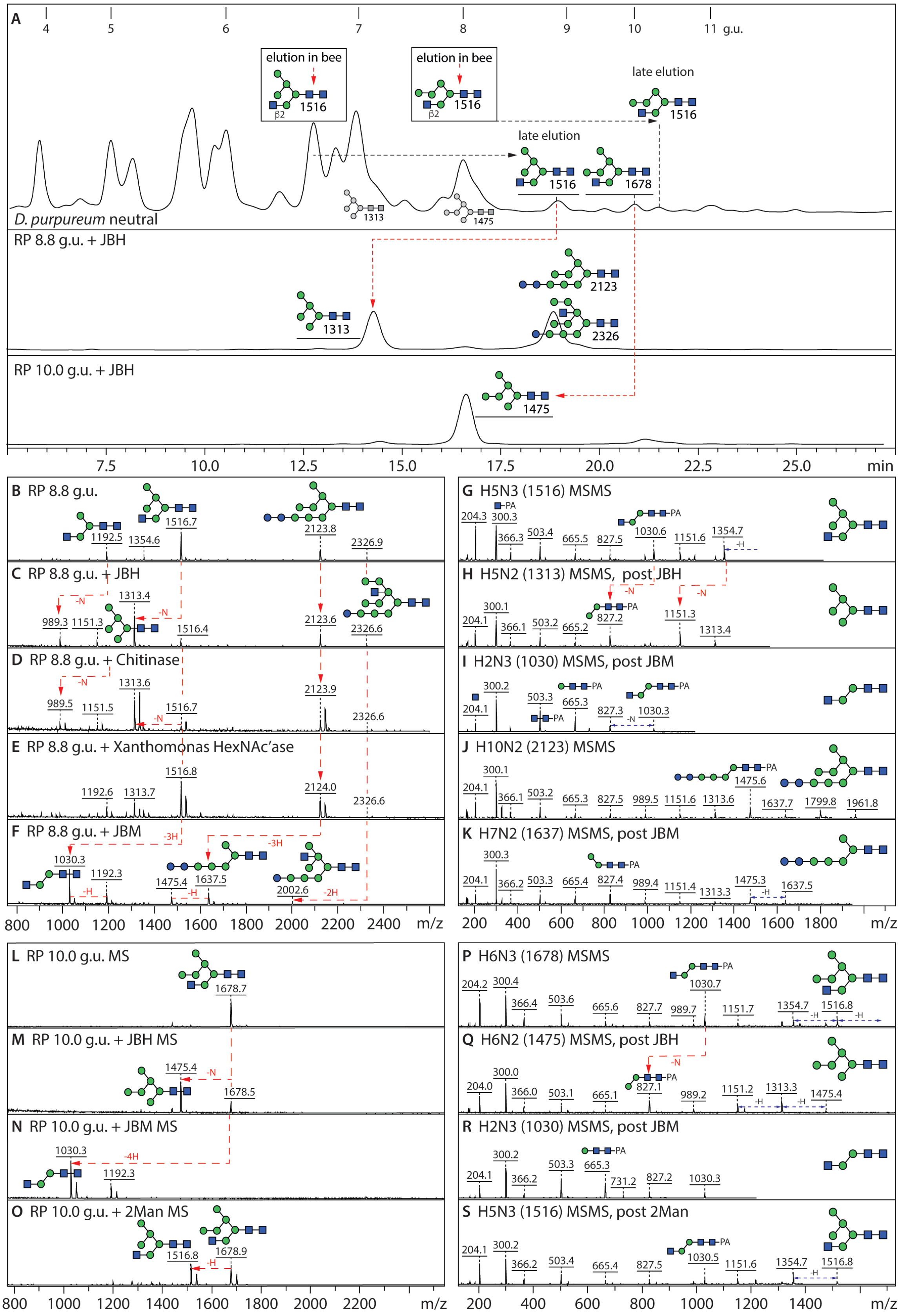

Figure S7: MALDI TOF MS/MS of RP-HPLC-separated galactosylated isomers from *D. purpureum*. The long linear galactose arm (β1,4-linkage; see also Figure 1 and 5 in the main text) was found on N-glycan structures with up to six galactose units. **(A-I)** The *m/z* 366, 528, 690, 852 and 1014 B fragment ions indicate 1, 2, 3, 4 and 5 hexoses linked to a HexNAc on the "lower" A arm (see inset in Figure 1). The PA-containing Y3α ions at *m/z* 1516, 1678 and 1840 also show the long arms (A-D), while the dominant Y3β or Y4 *m/z* 989, 1151, 1313 and 1475 ions, are indicative of the residual mannosylated structure, whereby the $Man_5GlcNAc_2$ can vary in the presence or absence of an α1,2-mannose residue as shown for *m/z* 2002 isomers (see Figure 5, also for results of galactosidase and hexosaminidase digestions). The different fragmentation patterns of the isomers (B, C; E, F; G, H, I) enable discrimination of the structures. **(J)** The fourth isomer shown of *m/z* 1678 is an intersected structure, eluting earlier than the galactosylated forms, whereas non-galactosylated isomers of m/z 1516 and 1678 with a non-reducing terminal GlcNAc are shown in Supplementary Figure 6.

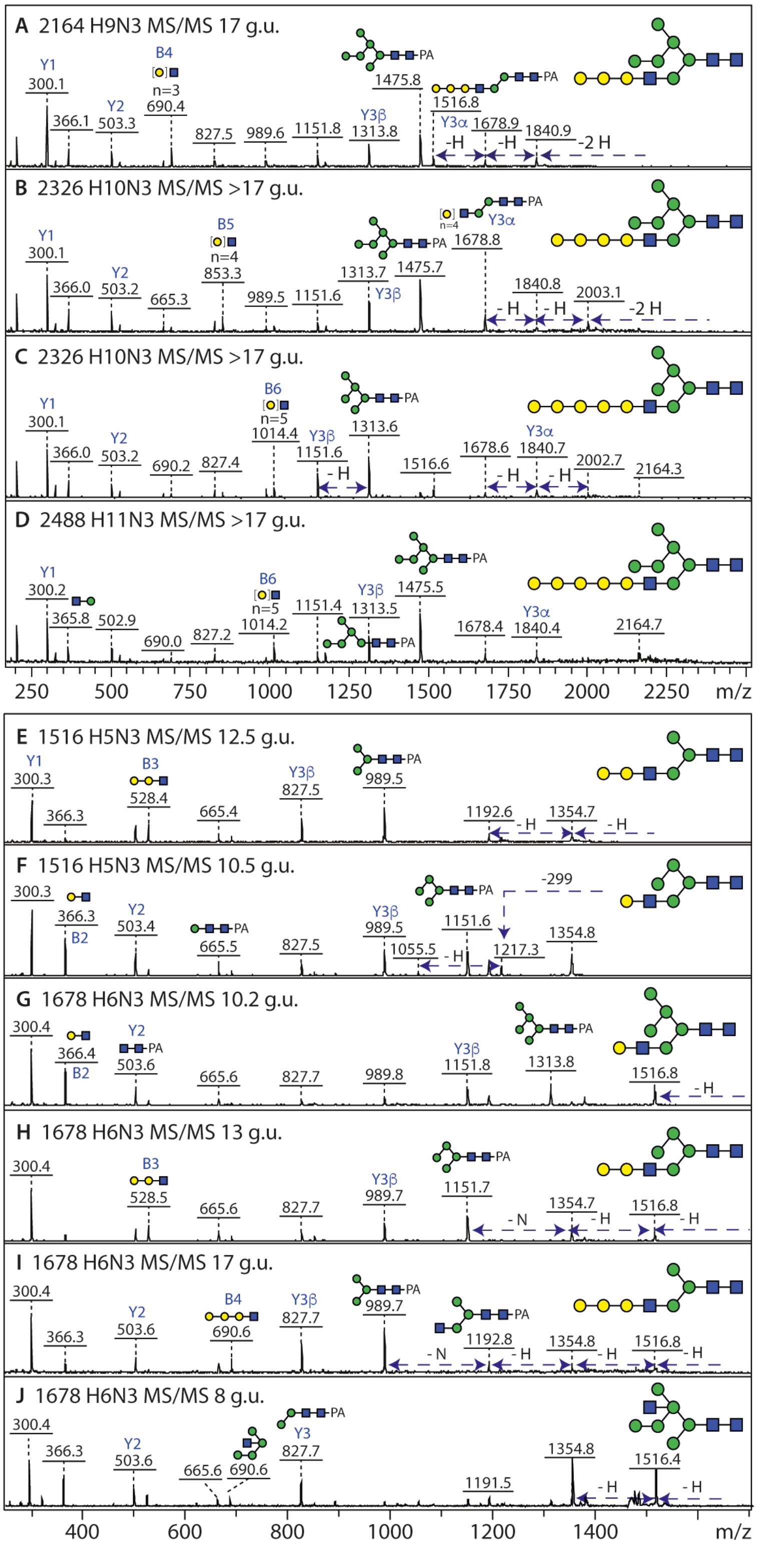

Figure S8: Core fucosylation in *D. purpureum*. (A-E) Two fractions of the *D. purpureum* neutral N-glycan pool eluting at 5.2 and 5.9 g.u. containing Hex$_{4\text{-}5}$HexNAc$_2$Fuc$_1$ (*m/z* 1297 and 1459) were subject to RP-HPLC and MS after hydrofluoric acid (HF) treatment. As observed in other *Dictyostelium* species and, *e.g.,* insect N-glycans, the core α1,3-fucose modification is observed with an early elution on the reverse phase system, correlates with an approximate 1:1 intensity ratio for the *m/z* 300 to *m/z* 446 fragments and is sensitive to HF, in contrast to core α1,6-fucose (see Ref. 68). The typical Y1 and Y2 fragment ions at *m/z* 446 and 649 are not visible after HF treatment indicative of the loss of core fucose (highlighted by red arrows; **G-L**). Subsequent α1,2-mannosidase digestion (**E, F and M**) aided definition of the 5.9 g.u. isomer of *m/z* 1459 Hex$_5$HexNAc$_2$Fuc$_1$ as having an unprocessed middle B arm; the later elution of the defucosylated form (8 g.u.) is also typical for glycans with an α1,2-mannose on the B-arm as compared to the more typical biosynthetic Man$_5$GlcNAc$_2$ structure (7.2 g.u.). A third m/z 1459 isomer, lacking the A arm, shifted from 5.7 to 7.6 g.u. after HF treatment (data not shown). Refer to Supplementary Figure 2 for MS/MS of late-eluting Man$_{5\text{-}6}$GlcNAc$_2$ isomers with an unprocessed B arm.

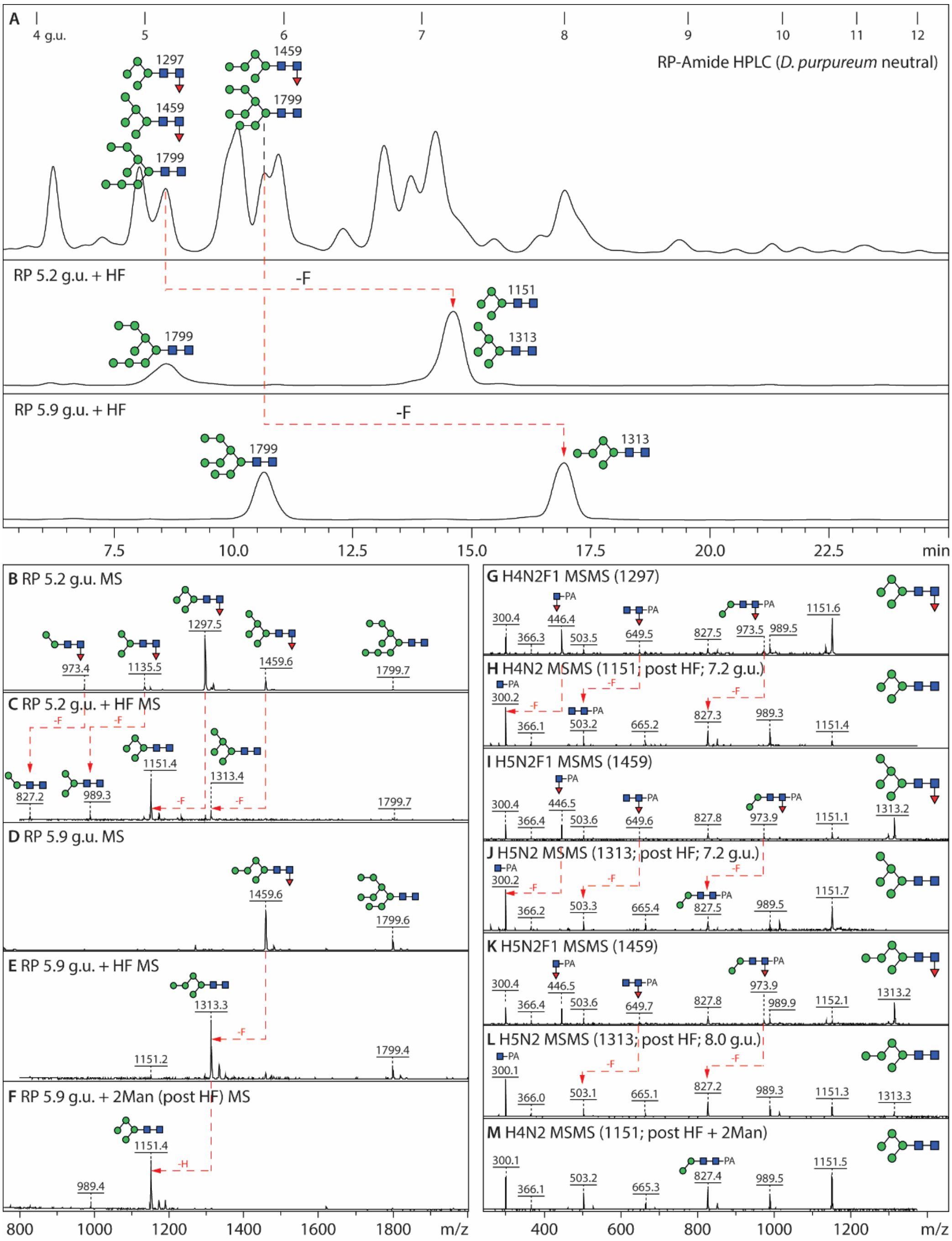

Figure S9: MALDI TOF MSMS definition of fucosylated isomers in *D. giganteum*. In contrast to *D. discoideum* and *D. purpureum* with only one core fucose modification, the neutral N-glycome of *D. giganteum* is highly fucosylated with single and multiple fucosylated antennae modifications (see also Figure 2A). The antennal fucose residues are to mannose either as mono- or difucose motifs, whereby the $Hex_6HexNAc_2Fuc_7$ structure with (*m/z* 2498; MS/MS shown in H) has the highest number of possible fucose modifications. Isomers of *m/z* 2351 $Hex_6HexNAc_2Fuc_6$ **(A-D)** and *m/z* 2059 $Hex_6HexNAc_2Fuc_4$ (6 to 15 g.u.) were identified at different elution times; these isomers have an equal number of fucose residues, but differ in their position within the "underlying" $Man_6GlcNAc_2$ structure. In the case of the $Hex_6HexNAc_2Fuc_4$ isomers, specific α1,2-mannosidase confirmed the unsubstituted "middle" B arm **(E-G)**. The Y3 fragments *m/z* 1119 and 1265 $Hex_2HexNAc_2Fuc_{2\text{-}3}$-PA are indicative of the lower arm modification and a Y1 *m/z* 446 fragment for core α1,3-fucose. The successive loss of the terminal fucose and hexose residues was observed from the parent ion in positive mode (highlighted in blue in all presented MS/MS data). $Hex_6HexNAc_2Fuc_7$ lost up to five fucose residues upon overnight incubation with bovine kidney α-fucosidase, which removes antennal fucose, but not core α1,3-fucose residues.

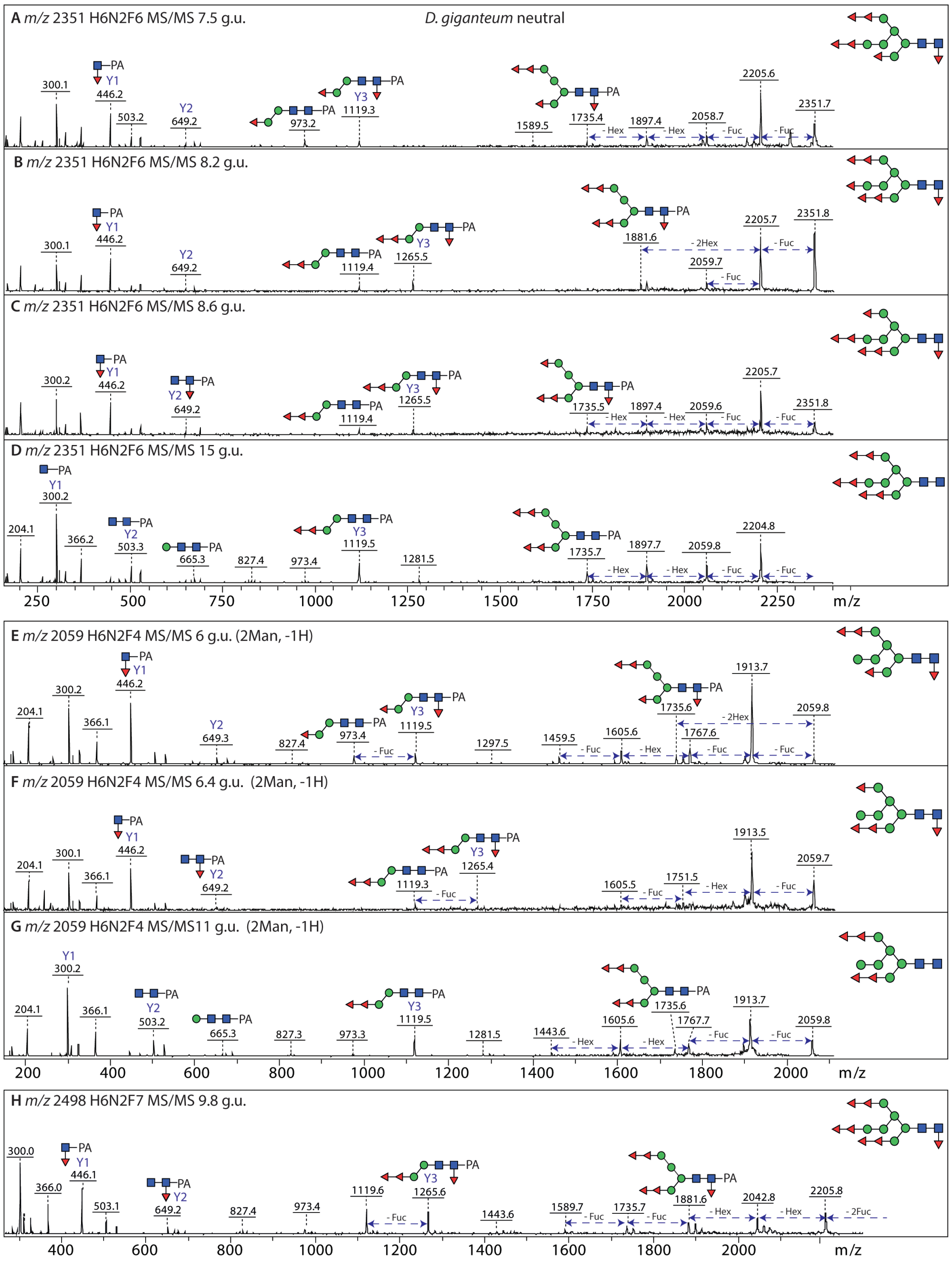

Figure S10: Sulphated anionic N-glycans in *D. purpureum*. (A, B) The 7 g.u. RP-HPLC anionic fraction of *D. purpureum* was analyzed with MALDI TOF MS and MS/MS in positive and negative ion modes. The mono sulphated glycans were detected in negative ion mode in the $[M\text{-}H]^-$ form, whereas multiply sulphated forms were observed as sodium adducts in the negative ion mode $[M\text{-}2H\text{+}Na]^-$ or $[M\text{-}3H\text{+}2Na]^-$ depending on the number of sulphate residues. In the positive ion mode, sulphation could not be detected, but in-source loss in positive mode facilitated MS/MS of the underlying structure (see inset). **(C, D)** Jack bean α-mannosidase and specific α1,2/3-mannosidase treatments resulted in losses of two, four or five hexose residues from the two glycans in the 7 g.u. fraction, indicative that the 'core' α1,6-mannose was sulphated. **(E)** Negative mode MALDI-TOF MS/MS of a sulphated intersected structure eluting at 8 g.u. shows an ^{0,2}A cross-ring fragment of the core GlcNAc, a set of serial losses from the core region and an *m/z* 241 B1 fragment indicative of sulphation of mannose; considering the pattern of mannosidase digestions of the non-intersected glycans, but the β1,4-linkage of the intersecting GlcNAc, the sulphation is assumed to be 1,2-linked to the α1,6 Man, i.e., not 6-linked to subterminal mannose as in *D. discoideum*. **(F-H)** Negative mode MALDI-TOF MS/MS of a trisulphated glycan (*m/z* 1919 as $[M\text{-}3H\text{+}2Na]^-$) and its two in-source fragments (*m/z* 1715 and 1817) indicating sulphation of a mannose residue close to the core chitobiose.

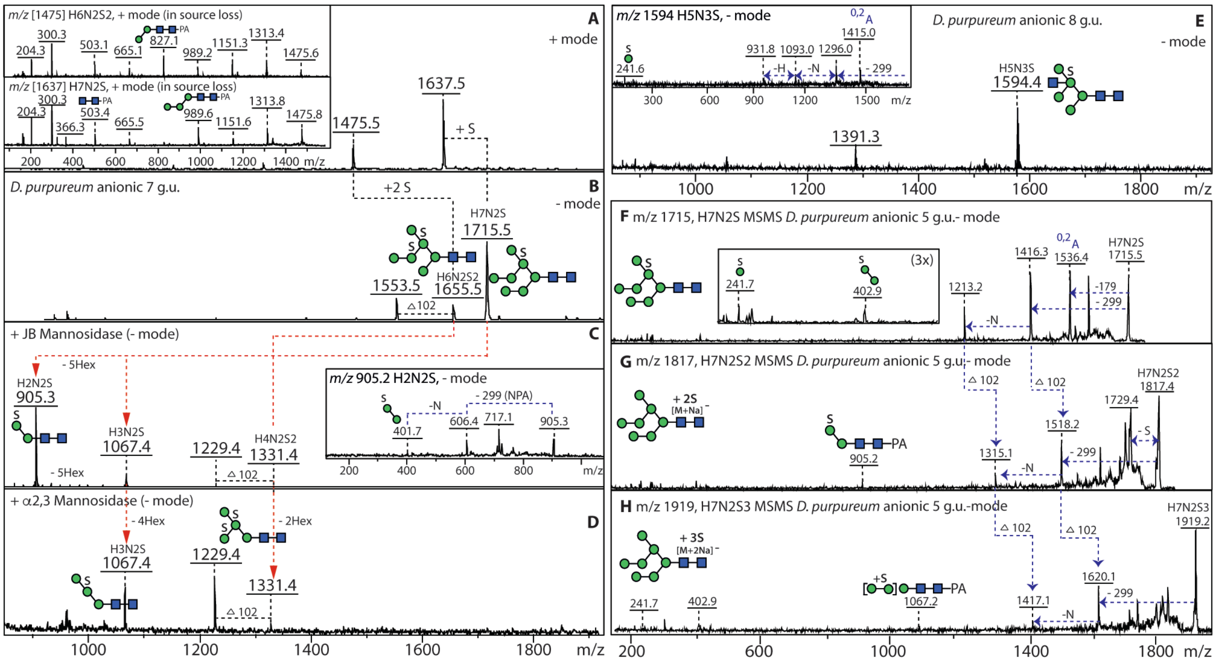

Table 1: Compositions of pyridylaminated N-glycans from *D. giganteum* and *D. purpureum* with the theoretical positive and (for anionic glycans) negative mode *m/z* values as well as the number of isomers per species or shared between the two species. For sulphated glycans (highlighted in grey), the positive mode *m/z* value in square brackets is that for the in-source fragment ion. Compositions are given in the form HxNyFzP/Me/S, whereby H is hexose, N N-acetylhexosamine, F fucose, P phosphate, Me methyl and S sulphate.

Composition	$[M+H]^+$	$[M-H]^-$	$[M-2H+ Na]^-$	$[M-3/4H+2/3 Na]^-$	Isomers		
					D. purp.	shared	D. gig.
**HN2F**	811.3				1	1	1
**H2N2**	827.3				1	1	1
**H2N2F**	973.4				1	1	1
**H3N2**	989.4				1	1	1
**H3N2S**	[989.4]	1067.3			1	1	1
**H3N2F**	1135.5				1	1	1
**H4N2**	1151.4				2	1	1
**H3N3**	1192.5				1		
**H3N2FS**	[1135.5]	1213.4					1
**H4N2S**	[1151.4]	1229.4			2	1	2
**H4N2P**	1231.4	1229.4			1		
**H4N2(PMe)**	1245.4	1243.4			1	1	1
**H4N2F**	1297.5				2	2	2
**H5N2**	1313.5				4	4	4
**H4N2S2**	[1151.4]		1331.4		2		
**H4N3**	1354.5				2	1	1
**H4N2FS**	[1297.5]	1375.4			1	1	2
**H5N2S**	[1313.5]	1391.4			4	3	3
**H4N2F(PMe)**	1391.5	1389.5			1		
**H5N2P**	1393.5	1391.5			2		
**H5N2(PMe)**	1407.5	1405.5			1	1	1
**H3N2F3**	1427.6						1
**H4N2F2**	1443.6						2
**H5N2F**	1459.6				3	3	3
**H6N2**	1475.6				4	3	3
**H5N2S2**	[1313.5]		1493.4		2		
**H4N3F**	1500.6						1
**H5N3**	1516.6				5	2	2
**H5N2FS**	[1459.6]	1537.5			1	1	2
**H6N2S**	[1475.6]	1553.5			4		
**H6N2P**	1555.5	1553.5			3	1	1
**H6N2(PMe)**	1569.5	1567.5			2	2	2
**H5N3S**	[1516.6]	1594.5			1		
**H5N2S3**	[1313.5]			1595.4	1	1	1
**H5N3P**	1596.5	1594.5			1		
**H5N2F2**	1605.6						2
**H6N2F**	1621.6				2	1	3

Composition	[M+H]$^{+}$	[M-H]$^{-}$	[M-2H+ Na]$^{-}$	[M-3/4H+2/3 Na]$^{-}$	Isomers		
**H7N2**	1637.6				4	3	3
**H6N2(PMe)S**	[1569.5]	1647.5			1	1	1
**H6N2S2**	[1475.6]		1655.5		1		
**H5N3F**	1662.6						3
**H6N3**	1678.6				7	2	2
**H5N2S4**	[1313.5]			1697.5	1		
**H6N2FS**	[1621.6]	1699.6			1	1	2
**H7N2S**	[1637.6]	1715.5			2		
**H6N2F(PMe)**	1715.6	1713.6			1	1	2
**H7N2P**	1717.6	1715.6			2	2	2
**H7N2(PMe)**	1731.6	1729.6			2	2	2
**H6N2(PMe)S2**	[1569.5]		1749.5		1	1	1
**H5N2F3**	1751.7						2
**H6N3S**	[1678.6]	1756.6			1		
**H6N2S3**	[1475.6]			1757.6	1		
**H6N3P**	1758.6	1756.6			2	1	1
**H6N2F2**	1767.7						4
**H7N2F**	1783.7				1	1	3
**H8N2**	1799.7				3	3	3
**H7N2(PMe)S**	[1731.6]	1809.5			1	1	1
**H7N2(PMe)P**	1811.6	1809.5			1	1	1
**H7N2S2**	[1637.6]		1817.5		2		
**H6N3F**	1824.7						2
**H7N2(PMe)2**	1825.6	1823.6			1	1	1
**H5N2F3S**	[1751.7]	1829.6					2
**H7N3**	1840.7				7	1	1
**H6N2F2S**	[1767.7]	1845.6					3
**H6N2(PMe)S3**	[1569.5]			1851.5	1	1	1
**H6N3S2**	[1678.6]		1858.6		1		
**H7N2FS**	[1783.7]	1861.6					1
**H7N2FP**	1863.6	1861.6			1		
**H7N2F(PMe)**	1877.6	1875.6			1	1	1
**H8N2S**	[1799.7]	1877.6			3	1	1
**H8N2P**	1879.6	1877.6			2		
**H7N2(PMe)PS**	[1811.6]	1889.6			1		
**H8N2(PMe)**	1893.6	1891.6			1	1	1
**H5N2F4**	1897.7						2
**H6N3FS**	[1824.7]	1902.7			1		
**H7N2(PMe)2S**	[1825.6]	1903.5			1	1	1
**H6N2F3**	1913.7						3
**H7N3S**	[1840.7]	1918.6			1		
**H7N2S3**	[1637.6]			1919.5	1		
**H7N3P**	1920.7	1918.6			1	1	2
**H7N2F2**	1929.7						1

Composition	[M+H]+	[M-H]-	[M-2H+ Na]-	[M-3/4H+2/3 Na]-	Isomers		
**H6N2(PMe)S4**	[1569.5]			1953.5	1		
**H9N2**	1961.7				3	2	2
**H8N2(PMe)S**	[1893.6]	1971.6					1
**H8N2(PMe)P**	1973.6	1971.6			1	1	1
**H5N2F4S**	[1897.7]	1975.7					1
**H8N2S2**	[1799.7]		1979.6		2		
**H7N3F**	1986.7						2
**H8N2(PMe)2**	1987.6	1985.6			2	1	1
**H6N2F3S**	[1913.7]	1991.7					2
**H8N3**	2002.7				7	1	1
**H7N2F2S**	[1929.7]	2007.7					1
**H7N2F2(PMe)**	2023.7	2021.7					1
**H9N2S**	[1961.7]	2039.7			2		
**H9N2P**	2041.7	2039.7			1		
**H5N2F5**	2043.8						2
**H8N2(PMe)PS**	[1973.6]	2051.6			1		
**H8N2(PMe)P2**	2053.6	2051.6			1		
**H9N2(PMe)**	2055.7	2053.7			1	1	1
**H6N2F4**	2059.8						3
**H8N2(PMe)2S**	[1987.6]	2065.6			1	1	1
**H8N2P(PMe)2**	2067.6	2065.6			1		
**H7N2F3**	2075.8						1
**H8N3S**	[2002.7]	2080.7			1		
**H8N2(PMe)3**	2081.6	2079.6			1	1	1
**H8N3P**	2082.7	2080.7			1	1	1
**H8N2F2**	2091.8						1
**H5N2F5S**	[2043.8]	2121.7					2
**H10N2**	2123.8				3	2	2
**H6N2F4S**	[2059.8]	2137.7					2
**H8N3F**	2148.8						2
**H9N2(PMe)2**	2149.7	2147.7			1		
**H7N2F3S**	[2075.8]	2153.7					1
**H9N3**	2164.8				6		
**H7N2F3(PMe)**	2169.8	2167.7					1
**H5N2F6**	2189.8						1
**H10N2S**	[2123.8]	2201.7			2		
**H6N2F5**	2205.8						3
**H7N2F4**	2221.8						1
**H9N3P**	2244.8	2242.7			1	1	1
**H6N2F5S**	[2205.8]	2283.8					1
**H11N2**	2285.8				2	1	1
**H9N3F**	2310.8						1
**H10N3**	2326.8				5	1	1
**H5N2F7**	2335.9						1

Composition	$[M+H]^+$	$[M-H]^-$	$[M-2H+Na]^-$	$[M-3/4H+2/3Na]^-$	Isomers		
**H6N2F6**	2351.9						4
**H11N2S**	[2285.8]	2363.8			2		
**H7N2F5**	2367.9						3
**H5N2F7S**	[2335.9]	2413.8					1
**H6N2F6S**	[2351.9]	2429.8					1
**H7N2F5S**	[2367.9]	2445.8					1
**H12N2**	2447.9				1	1	1
**H11N3**	2488.9				3	1	1
**H6N2F7**	2498.0						1
**H7N2F6**	2514.0						1
**H12N2S**	[2447.9]	2525.8			1		
**H6N2F7S**	[2498.0]	2575.9					1
**H12N3**	2650.9				2		
**H7N2F7**	2660.0						1
